# Ligand-Receptor Interactions of *Lamivudine*: A View from Charge Density Study and QM/MM Calculations

**DOI:** 10.3390/biomedicines11030743

**Published:** 2023-03-01

**Authors:** Alexander A. Korlyukov, Adam. I. Stash, Alexander R. Romanenko, Damian Trzybiński, Krzysztof Woźniak, Anna V. Vologzhanina

**Affiliations:** 1 A. N. Nesmeyanov Institute of Organoelement Compounds, Russian Academy of Sciences, 28 Vavilov St., Moscow 19334, Russia; 2 Biological and Chemical Research Centre, Department of Chemistry, University of Warsaw, Żwirki i Wigury 101, 02-089 Warszawa, Poland

**Keywords:** active pharmaceutical ingredients, HIV therapy, non-covalent interactions, *Lamivudine*, quantum theory ‘Atoms in Molecules’, QM/MM calculations, molecular Voronoi surface

## Abstract

The nature and strength of interactions for an anti-HIV drug, *Lamivudine*, were studied in a pure crystal form of the drug and the ligand–receptor complexes. High-resolution single-crystal X-ray diffraction studies of the tetragonal polymorph allowed the drug’s experimental charge density distribution in the solid state to be obtained. The QM/MM calculations were performed for a simplified model of the *Lamivudine* complex with deoxycytidine kinase (two complexes with different binding modes) to reconstruct the theoretical charge density distribution. The peculiarities of intramolecular interactions were compared with previously reported data for an isolated molecule. Intermolecular interactions were revealed within the quantum theory of ‘Atoms in Molecules’, and their contributions to the total crystal energy or ligand–receptor binding energy were evaluated. It was demonstrated that the crystal field effect weakened the intramolecular interactions. Overall, the energies of intermolecular interactions in ligand–receptor complexes (320.1–394.8 kJ/mol) were higher than the energies of interactions in the crystal (276.9 kJ/mol) due to the larger number of hydrophilic interactions. In contrast, the sum of the energies of hydrophobic interactions was found to be unchanged. It was demonstrated by means of the Voronoi tessellation that molecular volume remained constant for different molecular conformations (250(13) Å^3^) and increased up to 399 Å^3^ and 521(30) Å^3^ for the *Lamivudine* phosphate and triphosphate.

## 1. Introduction

**Figure 1:**
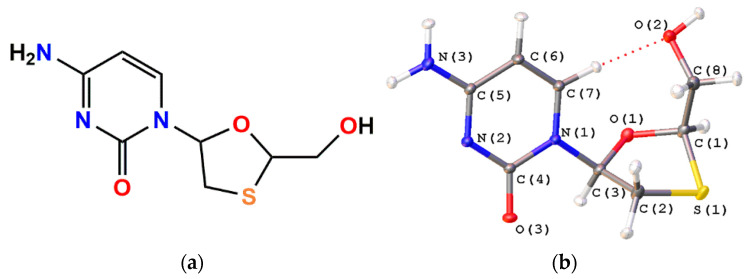
(**a**) Chemical formula of *Lamivudine*; and (**b**) its view in atomic displacement ellipsoids (50% probability) in the tetragonal polymorph II after the multipole refinement.

Nucleoside inhibitors of reverse transcriptase (NIRTs) are a great family of drugs for antiretroviral therapy for HIV. Among them, *Lamivudine* (the commonly adopted name is 3TC) is an old and widely used medication agent for the treatment of both HIV-1 [1] and HIV-2 [2]. Besides HIV, the activity of 3TC against hepatitis B [2] has been demonstrated. From a structural point of view, *Lamivudine*, as with most NIRTs, consists of the heterocyclic planar dihydroxypirimidine-2-one fragment and a modified cytidine (3-thiacytidine) moiety (Figure 1a). Both of its main structural parts contain additional donor and acceptor groups that can act as a donor and/or an acceptor of rather strong intermolecular interactions to form hydrogen bonds. In addition, two main structural fragments of 3TC are connected by an ordinary N-C bond, which allows for almost free rotation around this bond, so molecular conformation and shape can vary in order to realize various intermolecular interactions in the solid form or in a complex with the receptor. As a result, the above-mentioned features of 3TC have been utilized in many published articles [3,4,5,6,7,8,9,10,11,12,13,14,15,16,17,18,19,20,21,22,23,24,25,26] describing the preparation and crystal structures of different salts, polymorphs, and cocrystals with various co-formers. In total, the crystal structures of more than sixteen polymorphs, solvatomorphs, salts and cocrystals with various co-formers have been described up to date. On the contrary, in ligand–receptor complexes, 3TC is presented as *Lamivudine* triphosphate (3TC-3TP) almost everywhere, and there is only one example of a complex with protein where 3TC exists in its native chemically unmodified form.

**Scheme 1:**
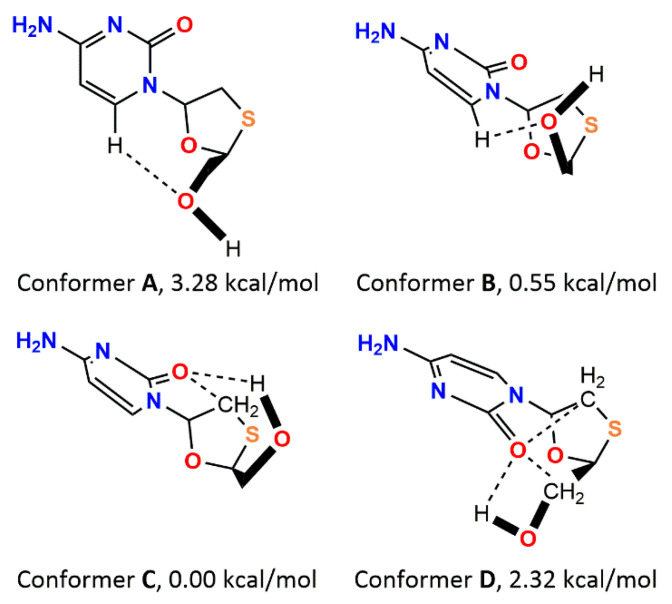
Schematic representation of intramolecular C-H…O and O-H…O interactions (dashed) in four stable conformations of 3TC, and their relative energies as obtained in ref. [29].

**Figure 2:**
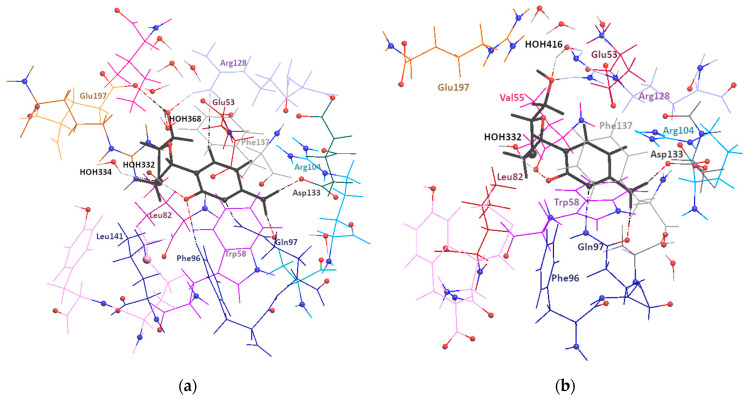
3D representation of 3TC molecule surrounding in complexes (**a**) A and (**b**) B of 2NOA structure [31]. 3TC molecule is black, and different residues are marked with different colors. H-bonds are shown with dashed lines. Blue and red spheres correspond to nitrogen and oxygen atoms of receptor.

As with many medication agents crucial for the therapy of HIV in many countries, 3TC became a subject of experimental [24] and computational [3,16,27,28] studies dedicated to discovering and characterizing the nature and strength of intermolecular interactions responsible for the stability of crystal structures, where the molecule of 3TC was present in its neutral or anionic forms. Early computational studies focused on the conformational analysis of 3TC and revealed its electronic structures [29]. It was demonstrated that the molecular conformation of an isolated 3TC molecule is characterized by the presence of four minima stabilized by weak intramolecular C-H…O (conformations **A** and **B**), and both strong O-H…O and weak C-H…O (conformations **C** and **D**) interactions (Scheme 1). The difference between the most stable and the less stable conformers is only 13.7 kJ/mol [29]. This value is comparable with the energy of one strong O-H…O hydrogen bond. Despite a detailed computational study of an isolated molecule of 3TC, information about the nature and the energy of intermolecular interactions formed by *Lamivudine* in the crystal or in ligand–receptor complexes is still incomplete. The most useful experimental method to remove such an incompleteness is an experimental high-resolution X-ray study, with subsequent topological analysis of the resulting charge density distribution in terms of the R. Bader’s quantum theory “Atoms in molecules” (AIM). Unfortunately, there are no charge density studies of 3TC-containing crystal structures published so far (charge density study of 3TC nitrate was mentioned in the book of abstract of ref. [30]; however, it seems to be still unpublished in scientific journals). Unfortunately, experimental charge density studies of the ligand–receptor complexes of 3TC are impossible, so quantum chemical studies of simplified systems are necessary to characterize adequately the charge density distribution in the region of the binding site including 3TC and surrounding amino acidic residues. Herein, we discuss an experimental charge density study of the tetragonal polymorph II of 3TC, especially the nature and energy of intermolecular interactions in its crystal packing. To compare the strength of intermolecular interactions with those in ligand–receptor complexes, quantum chemical calculations of simplified models based on the 2NOA structure [31] taken from PDB were carried out (Figure 2). The usage of Voronoi tessellation to the crystal structures of polymorphs, salts, solvatomorphs and ligand–receptor complexes of 3TC and 3TC-TP and the comparison with charge density and quantum chemical studies allowed us to verify how the results obtained for small molecules are applicable for biomolecules. The methods mentioned above were successfully applied for the study of intermolecular interactions in the crystal structures of the drug and ligand–receptor complexes of the imatinib salts [32], bicalutamide [33], and abiraterone acetate [34].

## 2. Materials and Methods

### 2.1. Dataset Collection, Reduction and Refinement

Single crystals were grown from commercially available tetragonal *Lamivudine* by slow evaporation from a water–ethanol mixture at −5 °C. A good-quality single crystal was selected from the precipitate and mounted on a glass capillary. X-ray diffraction data were collected at 100.0(2) K on an Agilent Super Nova diffractometer equipped with an Oxford Cryostream cooling device and Microfocus tube with a molybdenum anode (λ = 0.71073 Å). Omega and phi scans were carried out at several detector positions utilizing various exposure times to reach completeness and maintain sufficient redundancy at high diffraction angles. The measured intensities were integrated and corrected for absorption with CrysAlisPro software [35].

#### 2.1.1. IAM Refinement

Crystal structures were solved using the SHELXT software [36] and refined with SHELXL [37] and Olex2 programs [38] in an anisotropic approximation for non-hydrogen atoms. All hydrogen atoms were located on difference Fourier maps and refined in isotropic approximation as riding atoms with U_iso_ = 1.5U_eq_(O) for the hydroxy group and 1.2U_eq_(C,N) for other atoms. Convergency factors for this refinement are listed in Table 1. The resulting model was used as a starting point for the multipole refinement.

#### 2.1.2. Multipole Refinement

The charge distribution in 3TC was obtained using multipole formalism [39], as implemented in the XD2006 package [40], with core and valence electron density derived from wave functions fitted to a relativistic Dirac–Fock solution. In the first step, the scale factor was refined from all data. After that, high-order refinement (sin(θ)/λ > 0.7Å) of atomic positions and atomic displacement parameters incorporating all non-hydrogen atoms was employed, followed by refinement of hydrogen atoms positions, with C–H, N–H, and O–H distances fixed at values obtained from neutron diffraction [41]. The ADPs for hydrogen atoms were estimated using SHADE3 [42]. All non-hydrogen atoms were described at the octupolar level. For the H atoms, only the monopole population and the dipole populations were refined, while κ and κ’ were fixed at 1.2. Separate κ parameters were used for all non-hydrogen atoms; the κn’ (n = 1–4) parameters were also refined. During the final stage of the refinement, the multipole parameters, positions, and thermal parameters of all non-hydrogen atoms and monopoles were refined. All bonded pairs of atoms satisfied the Hirshfeld criterion. The experimental and refinement parameters are listed in Table 1. The molecular view is depicted in Figure 1b. Maps of residual electron density, analysis of the residual density according to Meindl & Henn [43,44], and the DRK-plot obtained via the WinGX suite [45] are also given in the Supporting Information (Figures S1–S3). For molecular graph see Figure S4. 

### 2.2. QM/MM Calculations of Receptor–3TC Complexes

To reveal the strength of interactions between 3TC and protein molecules, two simplified models were calculated. To construct these models, the structure of the deoxycytidine kinase complex with 3TC and ADP were used as a starting point. This structure contained two cavities with chemically unmodified 3TC molecules inside that could be considered as binding sites. The coordinates of 3TC molecules, surrounding peptide chains, and water molecules were extracted from the corresponding pdb file, and the positions of hydrogen atoms were recalculated. The number of amino acid residues and water molecules were sufficient to describe all interactions between 3TC and receptor molecules in selected binding sites. The next step was related to structure optimization. QM regions were chosen to consider all possible direct interactions between 3TC, amino acid residues, and water molecules. In the two models of receptor–3TC complexes (denoted as complex A and complex B), the sizes of QM regions used for optimization were 109 and 116 atoms, respectively (schematical representation is shown in Figure 2). MM regions of these models contained 2211 and 959 atoms, respectively. The following combination was applied to optimize the geometry of models: PBE/6-31G(d,p) for QM part and Amber force field for the rest of the amino acidic residues (ff19SB parameter set for MM part). Water molecules in the MM part and 3TC molecule were described using TIP3P and gaff2 parameters. All optimized configurations have been proven to be in the minima of energy by frequencies calculations. To calculate charge density functions, several amino acid residues that did not form any significant interactions with 3TC molecules but were disposed nearby were additionally included in the QM part. As a result, the charge density functions were calculated for clusters of 379 and 356 atoms (for complexes A and B, respectively). The geometry optimization, hessian and charge density calculations of complexes A and B were carried out using Gaussian09 software [46]. Charge density functions for complexes A and B were analyzed in terms of AIM theory [47], and the NCI method [48] using Multiwfn software [49]. Atomic coordinates of complexes A and B are listed in Supporting Information.

### 2.3. Voronoi Tessellation

For analysis of molecular Voronoi polyhedra, crystal structures of solids containing 3TC and H3TC^+^ were taken from the CSD, provided that there was no disorder for any of the atoms. The list of 19 solids is given in Appendix A. For these compounds, as well as for the optimized complexes A and B, the molecular Voronoi surfaces were analyzed, taking the coordinates of hydrogen atoms into account. For comparison with the ligand–receptor complexes containing 3TC and 3TC-TP, crystal structures from the PDB were taken (listed in Appendix A), and the coordinates of hydrogen atoms were added using the Hermes package [50]. Analyses of molecular Voronoi polyhedra, their volumes, and surfaces were carried out by means of the ToposPro package [51].

## 3. Results

### 3.1. Molecular Structure of 3TC in Crystal and Ligand-Receptor Complex

**Figure 3:**
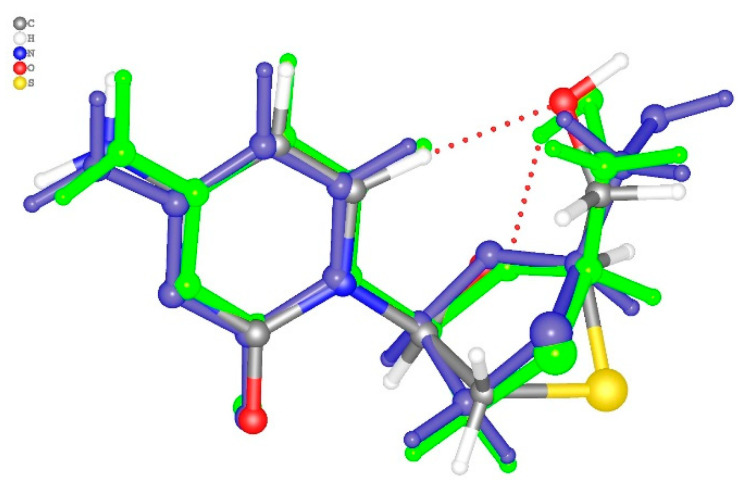
The superimposed structures of 3TC in crystal, complex A (blue color) and complex B (green color). Intermolecular C-H…O and O-H…O bonds are shown by the red dotted lines.

Fidanza et al. [29] mentioned that there exist four relatively stable *Lamivudine* conformations stabilized by different sets of intermolecular interactions. Depending on the values of torsion angles C7–N1–C3–O1 and O1–C1–C8–O2 in the tetragonal polymorph equal to 31.1(2) and 50.6(3)°, this conformation is close to conformation **A**, where the angles are equal to 14.3 and 47.3° (for conformation **B** the values are 46.3 and −65.2°). In the structure 2NOA complex, the refinement of *Lamivudine* was first carried out in the rigid body approximation [31]. Hence, two independent 3TC molecules are characterized by the same configuration with an O…O distance between the hydroxyl group and the oxygen atom of 3-thiacytidine moiety (2.715 Å). Moreover, the quality of X-ray data did not allow for precise localization of the positions of the hydrogen atoms, so it was impossible to obtain detailed experimental information about the H-bonds between 3TC and surrounding amino acid residues and water molecules. However, after QM/MM calculations, we obtained conformations of 3TC in complexes A and B with deoxycytidine kinase and ADP, slightly different from that in the crystal of tetragonal polymorph II (Figure 3). Their conformation is intermediate between the conformations **A** and **B**, with their torsion angles C7–N1–C3–O1 and O1–C1–C8–O2 in complexes A and B equal to, respectively, 0.2 and −76.7°, and −5.7 and −43.3°. The main differences in conformation are related to the deviation of the sulfur atom from the mean plane of the five-membered cycle and the position of the hydroxyl group. Notably, in complex A, the hydroxyl group does not participate in intramolecular bonds, while in complex B it forms a strong O-H…O bond with the oxygen atom of the five-membered cycle (O1…O2 distances are equal to 2.956 and 2.852 Å, respectively).

**Figure 4:**
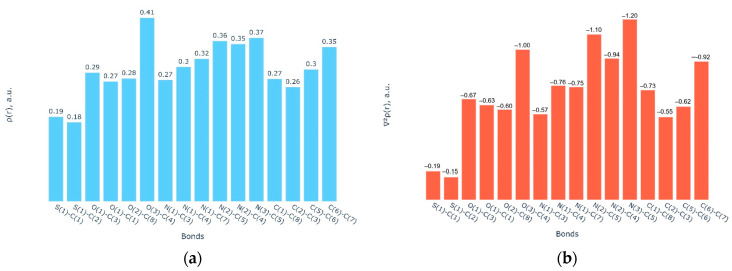
(**a**) The values of electron density and (**b**) its Laplacian in *bcps* in 3TC (atomic units).

The topological analysis of experimental electron density ρ(r) has shown the presence of bond critical points (*bcp* or CP(3,−1)) related to covalent bonds (Figure 4, Table S1). All bonds in 3TC are characterized by the negative value of Laplacian of electron density (∇^2^ρ(r)). The S-C bonds in 3TC have the least values (∇^2^ρ(r) comparable with the other bonds. A similar result was obtained in the charge density study of bicalutamide [33] and abiraterone acetate [34], published earlier.

It is worth noting that, despite minor differences in the conformation of *Lamivudine*, as visualized in Figure 3, these are supported by different sets of intramolecular interactions as obtained from the topological analysis of electron density ρ(**r**). Particularly, in the pure 3TC, a *bcp* is found for the C3-H3…O3 interaction between dihydroxypirimidine-2-one and oxatiolan-5-yl moiety, confirming that this molecule realizes the conformation **A**. The second intramolecular interaction is related to the bonding between the hydroxyl group and the dihydroxypirimidine-2-one fragment (C7-H7…O2). In the optimized 2NOA complexes with another conformation of the five-membered cycle, the conformation of 3TC is stabilized by intermolecular bifurcated bonding of the C-H group to the oxygen atom of the five-membered ring and a hydrogen atom of the CH_2_ fragment (complexes A and B), and an O-H…O bond and C-H…O=C interaction to, respectively, an oxygen atom and a CH_2_ group of the heterocycle (complex B).

Intramolecular bonds correspond to closed-shell interactions, so their energies can be evaluated with the correlation formula proposed by Espinosa et al. (EML, [52]). The energies of C-H…O bonds estimated using this formula in pure 3TC and its ligand–receptor complexes vary from −14.4 to −17.3 and from −16.8 to −17.1 kJ/mol. Using the results of the AIM study of the conformer **A** and EML formula, it is possible to evaluate the strength of similar C-H…O bonds in the conformer **A**. In an isolated conformer **A,** the strength of these C-H…O bonds becomes considerably larger (−21 and −25 kJ/mol). Such an overestimation of C-H…O bond strength is related to the effect of crystal packing. In ligand–receptor complexes, H…H interactions are also found to be as strong as −4.8–−9.0 kJ/mol. Anyway, the AIM study of 3TC has demonstrated the important role of the intramolecular bonding in stabilizing a given conformation and the fact that different sets of weak intramolecular interactions can stabilize similar conformations.

Considering that the 3TC molecule in both complex A and B realizes the conformation close to the experimental one in its tetragonal polymorph, their molecular shapes and molecular electrostatic potentials should also be similar. This makes a comparison of intermolecular interactions for each molecule of great interest.

### 3.2. Intermolecular Interactions of 3TC in Different Evironments

#### 3.2.1. H-Bonding Patterns in a Different Environment

Based on chemical composition, the *Lamivudine* molecule can form numerous hydrogen bonds. It contains two donor groups for H-bonding (the amine and hydroxyl) and five functional groups (oxygen atoms of the carbonyl O=C, hydroxyl OH, heterocycle, with heteroatoms N2_het_, O2_het_ and S) and potentially is able to act as an acceptor of H-bonding. Thus, pure *Lamivudine* can theoretically realize ten types of hydrogen bonds, listed in Table 2. However, the propensities of occurrences for these bonds can be ranked with good discrimination (the area under the ROC curve is equal to 0.89) to reveal the most and the least likely to occur, as described in refs. [53,54]. Surprisingly, the only expected intramolecular H-bond within this approach is the O-H…O2_het_ interaction observed in the complex B. The O-H…O=C interaction, which is expected to be present for two theoretically stable conformations proposed by Fidanza et al. [29], is unlikely to occur. For intermolecular interactions, the amino group is a better donor of H-bonds than the hydroxyl group; the carbonyl, hydroxyl, and N2_het_ atoms are good acceptors, while O2_het=_ and S are poor acceptors. This result is in accordance with the crystal structures of two known *Lamivudine* polymorphs, the tetragonal II and triclinic IV ones, where the most expected N-H…O=C interaction, and two highly likely (N-H…O(H) and O-H… N2_het_ for the tetragonal and N-H… N2_het_ and O-H…O=C for the triclinic polymorph) interactions are found.

**Figure 5:**
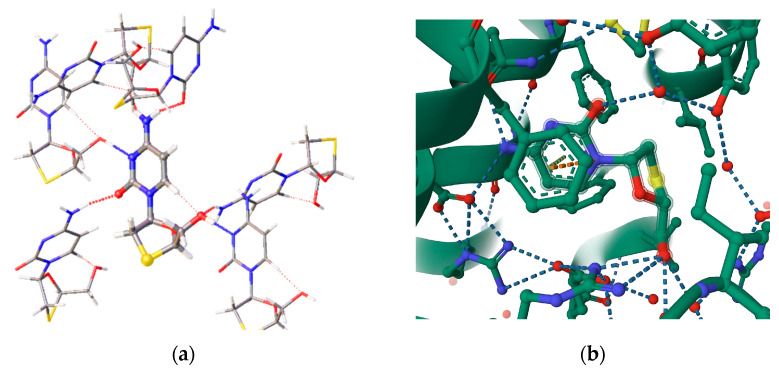
The H-bonding pattern of *Lamivudine* molecule in crystal structures of (**a**) pure 3TC (the tetragonal polymorph) and (**b**) 2NOA complex [31]. H-bonds are marked with dotted lines.

In co-crystals and ligand–receptor complexes, the presence of other donors and acceptors of H-bonding should be taken into account. While the amine and carbonyl groups can roughly mimic the amide fragment, the presence of water molecules and carboxylic groups from amino acids should also be considered. For the extended set of functional groups, the area under the ROC curve is 0.84, and the results of calculations indicate that, in general, carboxylate anions and water molecules are better donors of H-bonding. This is in accordance with experimentally observed crystal structures of *Lamivudine* hydrates, co-crystals with carboxylates, and H-bonding patterns of 3TC in ligand–receptor complexes (see Figure 5 for example).

It is worth mentioning that, in the 3TC–receptor complex, the values of hydrogen bonds between the 3TC and amino acid residues appear to be somewhat shorter than the corresponding ones in the crystal. For the strong N-H…O and O-H…O bonds, the values of N…O and O…O and of O…H and N…H distances are up to 0.4 Å shorter than in the crystal. In our opinion, the reason for this is related to the presence of the oxygen atoms with a high negative charge in the peptide chains. Indeed, in complex A, the hydroxyl group of 3TC participates in the strong hydrogen bond with the COO^−^ group of the glutamine residue (the O…H distance is equal to 1.622 Å). In complex B there is a strong N-H…O bond with the COO^−^ group of the asparagine residue (with the H…O distance equal to 1.694 Å). An analysis of interatomic distances in pure 3TC and complexes A and B can only give limited information related to the interactions between 3TC and peptide chains. The AIM studies of electron density function allow both strong and weak interactions to be revealed and a deeper insight gained into the molecular binding.

#### 3.2.2. Intermolecular Interactions and Their Sum Energies in a Different Environment

**Figure 6:**
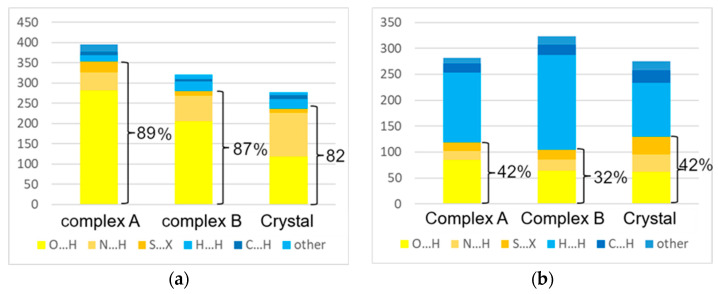
Contribution of hydrophobic (blue) and hydrophilic (yellow and orange) interactions to the (**a**) lattice energy and interaction energy in the tetragonal polymorph II and complexes A and B, and (**b**) to the surface of molecular Voronoi polyhedra.

In the crystal of *Lamivudine*, *bcps* for not only the above-mentioned strong hydrogen bonds but also for several weak C–H…O and C–H…H–C interactions are found. In contrast to the oxygen, nitrogen, and carbon atoms, the sulfur ones participate only in S…S, and S…C (S…π) interactions. Using the EML correlation, it is possible to evaluate the value of lattice energy as a sum of energies of all intermolecular interactions in the crystal. The strongest intermolecular interaction is the O2-H2…N2 bond; its energy is equal to −51.1 kJ/mol, which is much stronger than for any other interatomic interaction in the crystal of the tetragonal 3TC. Two other classic N-H…O bonds (N3-H3A…O3 and N3-H3B…O2) are much weaker, −17.9 and −14.3 kJ/mol, respectively. The S…S interaction appeared to be as strong as −10.3 kJ/mol, while the energy of other interactions with sulfur atoms does not exceed −7.4 kJ/mol. The energy of the H…H interactions varies from −1 to −3 kJ/mol. It should be noted that two intermolecular O2…O2 and O1…O3 interactions are found within the AIM approach; their energies are equal to −3.7 and −1.6 kJ/mol. The total value of the energies of all interatomic interactions evaluated via ELM correlation is −270.2 kJ/mol. The contribution of classic hydrogen bonds N-H…O and O-H…O appears to be uppermost. As a result, the contribution of hydrophilic interactions to the total crystal energy is equal to 82% (Figure 6a).

In the complexes A and B, the 3TC molecules form two strong N-H…O and O-H…O H-bonds (four and three, respectively, Table S2). Based on interatomic distances, their energy exceeds −30 kJ/mol, while in the crystal there is only one strong O2-H2…N2 H-bond. In addition, in the complexes A and B, there are also several hydrogen bonds with lower but considerable energy (from −10 to −30 kJ/mol). Similar to the crystal of the polymorph II, the 3TC molecule in complexes A and B also takes part in several C-H…O bonds, H…H and S…H and O…C interactions. The aromatic dihydroxypirimidine-2-one fragment of 3TC in complex B participates in a stacking interaction with the Ph group of phenylalanine. On the contrary, there are no stacking interactions in the crystal and complex A. In addition, an aromatic fragment of 3TC participates in C-H…π interactions with Ph groups of peptide chains. The energy of stacking interactions between dihydroxypirimidine-2-one and phenyl groups of PHE104 and PHE46 residues is equal to −14.4 and −7.5 kJ/mol (complexes A and B), respectively, which is negligible compared with all types of hydrogen bonds.

It is unsurprising that the value of the binding energies (−394.7 and −325.7 kJ/mol, respectively, for complexes A and B) exceeds that for the lattice energy of the 3TC tetragonal polymorph by 17 and 32%. The contributions of all the above-mentioned interactions to binding energy between 3TC and deoxycytidine kinase are shown in Figure 6a. The contribution of the O…H interactions due to the presence of strong N-H…O and O-H…O bonds (64 and 71% for complexes A and B, respectively) prevails over other types of interatomic interactions. Cumulative contributions of hydrophile H…O and N…H interactions in complexes A and B to binding energy are equal to 89 and 88%, correspondingly, so hydrophobic interactions of any type are responsible for only 11–12% of the binding energy (Figure 6). The integration of atomic basins and subsequent AIM calculation of atomic charges allows us to estimate the extent of charge transfer between 3TC and its environment. Despite the presence of strong hydrogen bonds and interactions with other types, the overall charge of the 3TC molecule becomes slightly positive (0.043 and 0.025 e). Most probably, the income of charge density caused by intermolecular interactions formed by sulfur atoms and negatively charged atoms of dihydroxypirimidine-2-one moiety almost compensates the charge density outcome due to the H-bonds between methylene, hydroxyl, and amine groups and surrounding electron-withdrawing atoms of water molecules and amino acid residues.

### 3.3. NCI Analysis of Intermolecular Interactions in Crystal and Complexes A and B

The non-covalent interaction (NCI) method gives information related to the nature of intra- and intermolecular interactions that is complementary to the AIM studies. The dimensionless reduced gradient function (RDG = |∇ρ(r)|/2 · (3π^2^)^1/3^ρ(r)^2/3^) derived from charge density is the primary quantity used in the NCI analysis. Isosurfaces of RDG function colored according to the sign of λ_2_ eigenvector can give insight into the nature of intermolecular interaction (attractive/repulsive) and reveal the area related to interatomic contacts.

**Figure 7:**
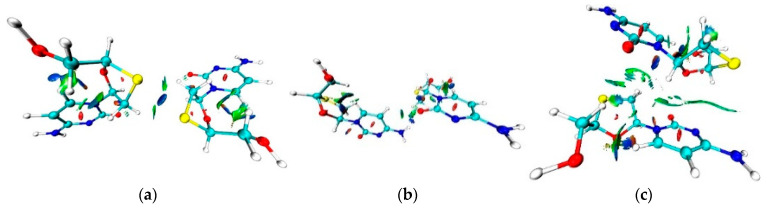
The isosurfaces of RDG function (0.35 a.u.) colored according to the λ_2_ eigenvector (−0.015–0.015 a.u.). Negative values are blue-colored while positive ones are red-colored. The figure depicts the attractive (**a**) S…S interaction, (**b**) N-H…O and intramolecular C-H…O bond, (**c**) H…H and C-H…π interactions.

The exploration of isosurfaces of RDG function in the crystal also revealed the presence of several rather strong H-bonds. The maxima of RDG function in the regions of N-H…O and O-H…O, bonds and S…S interaction are small, oblate, blue-colored spots that are indicative of strong attractive nature (Figure 7a,b). On the contrary, C-H…O, H…H, S…H and C-H…π interactions are characterized by a value of sign(λ_2_) that is close to zero (fuzzy green- or green-blue colored regions, Figure 7c) that clearly reveals its van-der-Waals nature.

**Figure 8:**
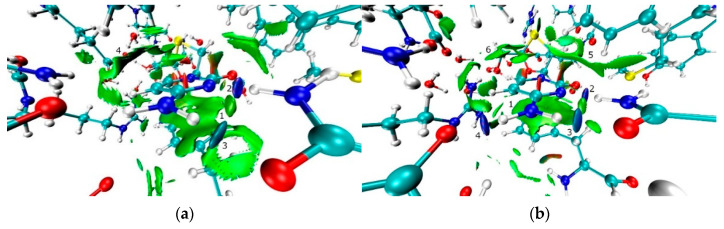
The isosurfaces of RDG function (0.35 a.u.) colored according to the λ_2_ eigenvector (−0.02–0.04 a.u.) in the region of dihydroxypirimidine-2-one moiety of complexes A and B ((**a**) and (**b**), respectively). Negative values are blue-colored while positive ones are red-colored. The stacking interactions appear near point 1, while strong N-H…N and N-H…O bonds are close to points 2 and 3, respectively, (**a**) or 2, 3, 4 (**b**). The C-H…π interactions are visualized at points 4 (**a**) or 5, 6 (**b**).

**Figure 9:**
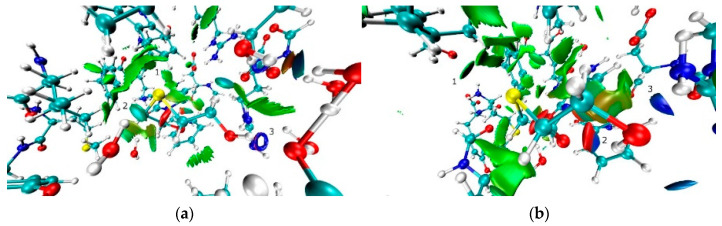
The isosurfaces of RDG function (0.35 a.u.) colored according to the λ_2_ eigenvector (−0.02–0.04 a.u.) in the region of the 3-thiacytidine moiety of complexes A and B ((**a**) and (**b**), respectively). The negative values are blue-colored, while positive ones are red-colored. The C-H…S interactions are depicted at point 1, while the O-H…S interaction (**a**) and intramolecular O-H…O bond (**b**) are depicted at point 2. Near point 3, a strong O-H…O bond with COO- moiety of an amino acid (**a**) or N-H…O bond (**b**) is observed.

Similarly, the analysis of RDG function for the crystalline complexes A and B clearly demonstrates the attractive character for almost all strong O-H…O and N-H…O bonds (Figure 8 and Figure 9). The first of two exceptions is a strong O-H…O bond between the hydroxyl group of 3TC and COO- moiety of glutamine residue while the second is an intramolecular O-H…O bond in 3TC. The corresponding maxima of RDG function are characterized by the presence of regions with positive values of λ_2_ (red color). The reason can be related to steric repulsion between negatively charged oxygen atoms. The stacking, C-H…π, O-H…S and C-H…S interaction can be characterized as van-der-Waals interactions. Despite rather high surface areas, they are characterized by values of λ_2_ that are close to zero. The character of O-H…S and C-H…S interactions formed by 3TC differ considerably from those formed by sulfur atoms of cysteine. In the latter case, almost all bonds can be characterized as attractive ones [55].

### 3.4. Contribution of Various Types of Intermolecular Interactions to the Voronoi Surface of Lamivudine and Its Derivatives

Within the Voronoi tessellation of crystal space, all points within an atomic domain are closer to the inner atom than to any external one. A molecular Voronoi polyhedron (MVP) consists of atomic Voronoi polyhedra constructing the molecule [56]. The faces of atomic Voronoi polyhedra form the molecular Voronoi surface, and a pair of atoms that shares a molecular surface is supposed to form an intermolecular interaction. The MVP represents well molecular volumes and networks of intermolecular interactions in crystals of small molecules [34,57,58,59,60,61], compared with the AIM approach. It has also been applied to compare *Imatinib* interactions in small molecules and ligand–receptor complexes [32] and for analysis of protein volumes and packing [62,63,64], and for interactions between macromolecules [65,66]. However, a comparison of MVP and AIM for ligand–receptor complexes has not been carried out to our knowledge.

The number of facets on atomic Voronoi polyhedra exceeds the number of intermolecular interactions. Thus, the faces that correspond to indirect interactions (the line between a pair of atoms does not intersect their common face of a Voronoi polyhedron) or to direct interactions with small solid angles Ω are supposed to be non-binding or forced interactions. It has previously been demonstrated that Ω > 10, 10, and 5% allows the majority of strong and weak hydrogen bonds, agostic interactions, and halogen bonds to be revealed [34,57,58,59,60,61]. We carried out a similar analysis for the *Lamivudine* molecule in the crystal and two optimized ligand–receptor complexes. The results are summarized in Table 3. Similar to previously reported results, one can conclude that the indirect interactions are non-bonding even when the corresponding Ω value is large (*bcps* were found only for 2 of 4525 indirect neighbors). Secondly, the Ω value that allows revealing bonding interactions depends on the type of the connected atoms. For both strong and weak hydrogen bonds, Ω > 5% reveals the majority of such interactions with a minimum of false positive and false negative interactions. Dihydrogen bonds Ω > 10% should be used to decrease the number of false positive H…H interactions. It is worth mentioning also that similar Ω values can be used for both single crystals in small molecules and ligand–receptor complexes.

In Table 3, both intra- and intermolecular interactions are summarized. However, it can be mentioned that in ligand–receptor complexes the overall number of hydrogen bonds is higher than that in pure *Lamivudine* due to a different molecular environment. The number of H-bond donors in ligand–receptor complexes is higher. Thus, the oxygen and sulfur atoms of the *Lamivudine’* heterocycle are also able to act as acceptors of H-bonding. That is why no S…S bonding is observed in ligand–receptor complexes. The contribution of various types of intermolecular interactions to the Voronoi molecular surface can be compared with that of the sum energy of molecular bonding. These data are visualized in Figure 6b.

First of all, it should be mentioned that, within the Voronoi tessellation, there are bonding, non-bonding, and forced contacts for the molecular surface. Thus, besides bonding interactions revealed within the AIM approach, some surface goes to N…O, O…O, and S…N interactions, although their contribution to the total molecular surface is very low (Table S3). Another important difference between the results of the Voronoi tessellation and the AIM theory is that the contribution of hydrophilic interactions to the molecular surface is nearly two times lower (c.a. 40% vs. 85%) than the contribution to the sum energy of molecular interactions. This fact results from an equal division of crystal space between two atoms with non-equivalent electronegativity in the Voronoi approach. In fact, a zero-flux surface is shifted to one of them. Besides, for complex B with elongated interatomic distances and probably voids with missing atoms and groups of atoms, the results of the Voronoi tessellation are not highly reliable—this can explain both the enlarged molecular volume and surface, and the fact that two *bcps* in this complex correspond to non-direct interactions. Nevertheless, some regularities concerning the role of different types of interactions remain common for both models: (i) the contribution of the hydrophilic interactions to the molecular surface remains nearly constant (for complex A and crystal), (ii) the contribution of the H…O interactions to the molecular surface and sum energy is higher than that of H…N interactions, and in ligand–receptor complexes the former are more frequently observed.

**Figure 10:**
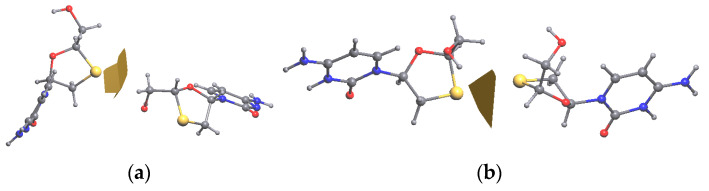
Intermolecular S…H and S…S. interactions and the corresponding surface of Voronoi polyhedra in crystals of (**a**) 3TC·0.5H_2_O [18] and (**b**) H3TC(C_2_O_4_)·0.5H_2_O [24].

Qualitative and semiquantitative correlation for these two models also allows the use of the Voronoi tessellation for analysis of intermolecular interactions in other solids and ligand–receptor complexes. We considered 19 fully-ordered solids containing the 3TC neutral molecule or the H3TC^+^ cation (including the tetragonal polymorph) and four ligand–receptor complexes with 3TC, 3TC-TP or 3TC-3TP (TP and 3TP denote the phosphate or triphosphate group), where no empty space is present near the ligand. The molecular volume and surface for this molecule remain constant for different conformations, environments (both in crystals and complexes), and compositions (for 3TC and H3TC^+^), and are equal to 250(13) Å^3^ and 270(11) Å^2^. These values increase in *Lamivudine* phosphate (399 Å^3^ and 398 Å^2^) and triphosphate (521(30) Å^3^ and 502(20) Å^2^). The contribution of hydrophilic interactions to the molecular Voronoi surface of *Lamivudine* forms varies from 36 to 61%. For complexes of 3TC-TP and 3TC-3TP, the area that goes to hydrophilic interactions increases (173 and 265(9) Å^2^ compared with 127(14) Å^2^), but the percentage remains nearly unchanged (43% and 53(3)% compared with 47(5)%). Analysis of unusually high contributions of some interactions to the molecular surface allows revealing, for example, of chalcogen bonds or strong S…H interactions (Figure 10).

## 4. Conclusions

Peculiarities of intermolecular interactions of the *Lamivudine* molecule were studied using a combination of experimental and theoretical approaches. The likelihood of formation of strong hydrogen bonds decreases from amine- to hydroxide group as donors and from O=C to OH, N_het_, O_het_ and to S_het_ atoms as acceptors of H-bonding. This fact is in accord with experimentally observed structures, where O_het_ and S_het_ atoms take part in H-bonding only at ligand–receptor complexes, and with the energies of hydrogen bonds, which fall from O–H…N (−51.1 kJ/mol, crystal) to N–H…N (−41.6 kJ/mol, complex), N–H…O (vary from −14.3 to −35.9 kJ/mol, crystal; vary from −28.9 to −57.6 kJ/mol, complex), O-H…O (vary from −7.9 to −26.2, complex), and O-H…S (−15.7 kJ/mol, complex). Similar types of interactions in complexes are, in general, stronger than in pure solids. The increased number of H-bond donors in the molecular environment also results in an increased sum of molecular energy from the ligand–receptor complexes (320.1–394.8 kJ/mol) to the crystal (276.9 kJ/mol). This increase comes mainly from hydrophilic interactions, as both their sum energy and contribution to the overall energy of interactions increases.

For comparison of *Lamivudine’s* intermolecular interactions in a series of compounds, the Voronoi tessellation of crystal space was applied. It is demonstrated for the first time that, within this approach, bonding and non-bonding interactions can be revealed not only for solids but also for ligand–receptor complexes. Particularly, no *bcps* are found for the non-direct contacts in terms of the Voronoi tessellation, while direct interactions with the solid angles Ω = 5, 5 and 10% allow for revealing 79, 91 and 77% of strong, weak hydrogen bonds and dihydrogen interactions with a minimum of false positive results. These Ω values coincide with previously reported ones for solids containing small molecules. Analysis of 23 solids comprising 44 independent *Lamivudine* molecules or their derivatives within the Voronoi approach demonstrates that molecular volume remains constant for different molecular conformations (250(13) Å^3^) and increases up to 399 Å^3^ and 521(30) Å^3^ for *Lamivudine* phosphate and triphosphate, respectively. The overall area of hydrophilic interactions also increases from pure *Lamivudine* to phosphate and triphosphate.

## Figures and Tables

**Table 1:** Details of experiment and refinement of the tetragonal polymorph II of *Lamivudine*.

Parameter	3TC	
	IAM ^1^ on F^2^	MM ^1^ on F/F^2^
Chemical formula	C_8_H_11_N_3_O_3_S	
Mr	229.26	
Crystal system, space group	P4_3_2_1_2	
a, c (Å)	8.65125(3), 26.39191(18)	
V (Å^3^)	1975.280(19)	
Z	8	
μ (cm^−1^)	0.319	
Crystal size, mm	0.479 × 0.379 × 0.272	
T_min_, T_max_	0.335, 1.000	
No. of meas., indep. and obs. [I > 2σ(I)] refls	275,252, 11,917, 10,708	
R_int_	0.0753	
(sin θ/λ)_max_ (Å^−1^)	1.13	
Flack	0.001(9)	
No. of parameters	180	796
Goodness-of-fit	1.033	1.097
R_1_ [all data]	0.0305	0.0218/0.0187
wR_2_ [all data]	0.069	
R_1_ [*I > 2σ(I)*]	0.0253	0.0154/0.0178
wR_2_ [*I > 2σ(I)*]	0.0671	0.031/0.029
Δρ_max_, Δρ_min_ (e Å^−3^)	0.47, −0.25	0.24, −0.25

^1^ IAM—independent atom model refinement; MM—multipole model refinement.

**Table 2:** Propensities of intermolecular H-bonding in 3TC-containing solids.

Donor	Acceptor	Propensity ^1^	Observed in Solid 3TC ^2^	Observed in CSD ^3^	Observed in PDB
Amine NH_2_	^−^OOC	-/0.94		Yes	Yes
	H_2_O	-/0.62		Yes	Yes
	O=C	0.46/0.52	Tetr, Tric	Yes	Yes
	HO	0.42/0.50	Tetr	Yes	Yes
	N2_het_	0.40/0.48	Tric	Yes	Yes
	O2_het_	0.14/0.19		Yes	
	S_het_	0.06/0.11			
OH	^−^OOC	-/0.90		Yes	
	H_2_O	-/0.48			Yes
	O=C	0.41/0.39	Tric	Yes	
	OH	0.38/0.40		Yes	
	N2_het_	0.36/0.38	Tetr	Yes	
	O2_het_	0.12/0.13			
	S_het_	0.05/0.06			
H_2_O	^−^OOC	-/0.95		Yes	
	H_2_O	-/0.65			
	O=C	-/0.60		Yes	Yes
	HO	-/0.55		Yes	
	N2_het_	-/0.55		Yes	
	O2_het_	-/0.23			
	S_het_	-/0.12			Yes

^1^ Data at absence and presence of water molecules and carboxylic groups are divided by slash. ^2^ Data for the tetragonal II (Tetr) and triclinic IV (Tric) polymorphs are given. ^3^ Data for co-crystals, salts, and solvates.

**Table 3:** The dependence between “direct” interactions with high solid angles and the presence of a *bcp* for these interactions ^1^.

Ω, %	Interaction ^2^	False Negative	Positive	False Positive
Tetragonal polymorph				
5	D–H…A	0	4	0
	C–H…A	1	7	0
	H…H	0	7	7
	C–H…π	1	1	0
	Other	0	1	0
	Overall	2	19	7
10	D–H…A	1	3	0
	C–H…A	5	3	0
	H…H	4	10	0
	C–H…π	2	0	0
	Other	1	0	0
	Overall	13	15	0
Complex A				
5	D–H…A	5	16	3
	C–H…A	0	5	3
	H…H	0	9	28
	C–H…π	0	2	0
	π…π	3	0	0
	Overall	8	32	34
10	D–H…A	12	8	1
	C–H…A	2	2	3
	H…H	1	7	4
	C–H…π	2	3	0
	π…π	3	0	0
	Overall	20	20	8
Complex B				
5	D–H…A	3	15	3
	C–H…A	1	5	3
	H…H	0	7	27
	C–H…π	2	1	1
	π…π	1	0	0
	Overall	7	28	0
10	D–H…A	9	9	2
	C–H…A	3	3	0
	H…H	2	3	9
	C–H…π	4	1	0
	π…π	1	0	0
	Overall	19	16	11

^1^ False negative, positive and false positive denote, respectively, interactions for which (i) a *bcp* is found, but the Ω angle is below the reference value, (ii) the presence of a *bcp* for contact with the angle above the reference Ω value, and (iii) contact with the Ω angle above the reference value, but without a *bcp*. ^2^ Strong donors (O, N) and acceptors (O, N, S) of H-bonding are denoted as A and D, respectively.

## Data Availability

The X-ray data are available at CCDC under ref. code CCDC 2239677.

## Supplementary Materials

The following supporting information can be downloaded at: https://www.mdpi.com/article/10.3390/biomedicines11030743/s1, Figure S1: DRK plots; Figure S2: residual density map; Figure S3: fractal dimension vs. residual density plot; Figure S4: molecular graph of *Lamivudine*; atomic coordinates of complexes A and B; Table S1: descriptors of *bcp* in polymorph II of *Lamivudine*; Table S2: descriptors of *bcp* in complexes A and B; Table S3: descriptors of MVP in previously reported complexes; CIF and FCO files.

## Appendix A

CSD codes taken for analysis of *Lamivudine* interactions and molecular Voronoi polyhedra: COWSUD, COWTAK, IGAVEU, LASZAI, LASZEM01, LICVEB, LIGWUV, NUWKEX, QUWXOW, RIBFIT, RIBFOZ, RUKHAG, RUKHAG01, UYEYEE, VAWPIT, VISVOK, VISVUQ, VISWAX, WOMHEM. PDB codes taken for analysis of molecular Voronoi polyhedra: 2NOA, 4QWD, 5TB8, 5TBC.
