# Supplementary material for: Ligand-Receptor Interactions of Lamivudine: A View from Charge Density Study and QM/MM Calculations
Source: Biomedicines. 2023 Mar 1;11(3):743. doi: 10.3390/biomedicines11030743 (PMC10045540; doi:10.3390/biomedicines11030743)
Supplement: Supplementary file 1 [file biomedicines-11-00743-s001.zip › biomedicines-2229318-supplementary.pdf]

Supporting Information for article:

Ligand-Receptor Interactions of *Lamivudine*: A View from Charge Density Study and QM/MM Calculations

Alexander A. Korlyukov [1], Adam. I. Stash [1], Alexander O. Romanenko [1], Damian Trzybiński [2], Krzysztof Woźniak [2] and Anna V. Vologzhanina [1,*]

[1] A. N. Nesmeyanov Institute of Organoelement Compounds, Russian Academy of Sciences, 28 Vavilov St., Moscow 19334, Russia; alex@xrlab.ineos.ac.ru (A.A.K.); astas@yandex.ru (A.I.S.); alex070401@gmail.com (A.R.R.)

[2] Biological and Chemical Research Centre, Department of Chemistry, University of Warsaw, Żwirki i Wigury 101, 02-089 Warszawa, Poland; dtrzybinski@cnbc.uw.edu.pl (D.T.); kwozniak@chem.uw.edu.pl (K.W.)

* Correspondence: vologzhanina@mail.ru

Citation: Korlyukov, A.A.; Stash, A.I.; Romanenko, A.R.; Trzybiński, D.; Woźniak, K.; Vologzhanina, A.V. Ligand-Receptor Interactions of *Lamivudine*: A View from Charge Density Study and QM/MM Calculations. *Biomedicines* **2023**, *11*, x. https://doi.org/10.3390/xxxxx

Academic Editor: Eugenia Pechkova

Received: 3 February 2023
Revised: 21 February 2023
Accepted: 23 February 2023
Published: 28 February 2023

Copyright: © 2023 by the authors. Licensee MDPI, Basel, Switzerland. This article is an open access article distributed under the terms and conditions of the Creative Commons Attribution (CC BY) license (https://creativecommons.org/licenses/by/4.0/).

DRK plots

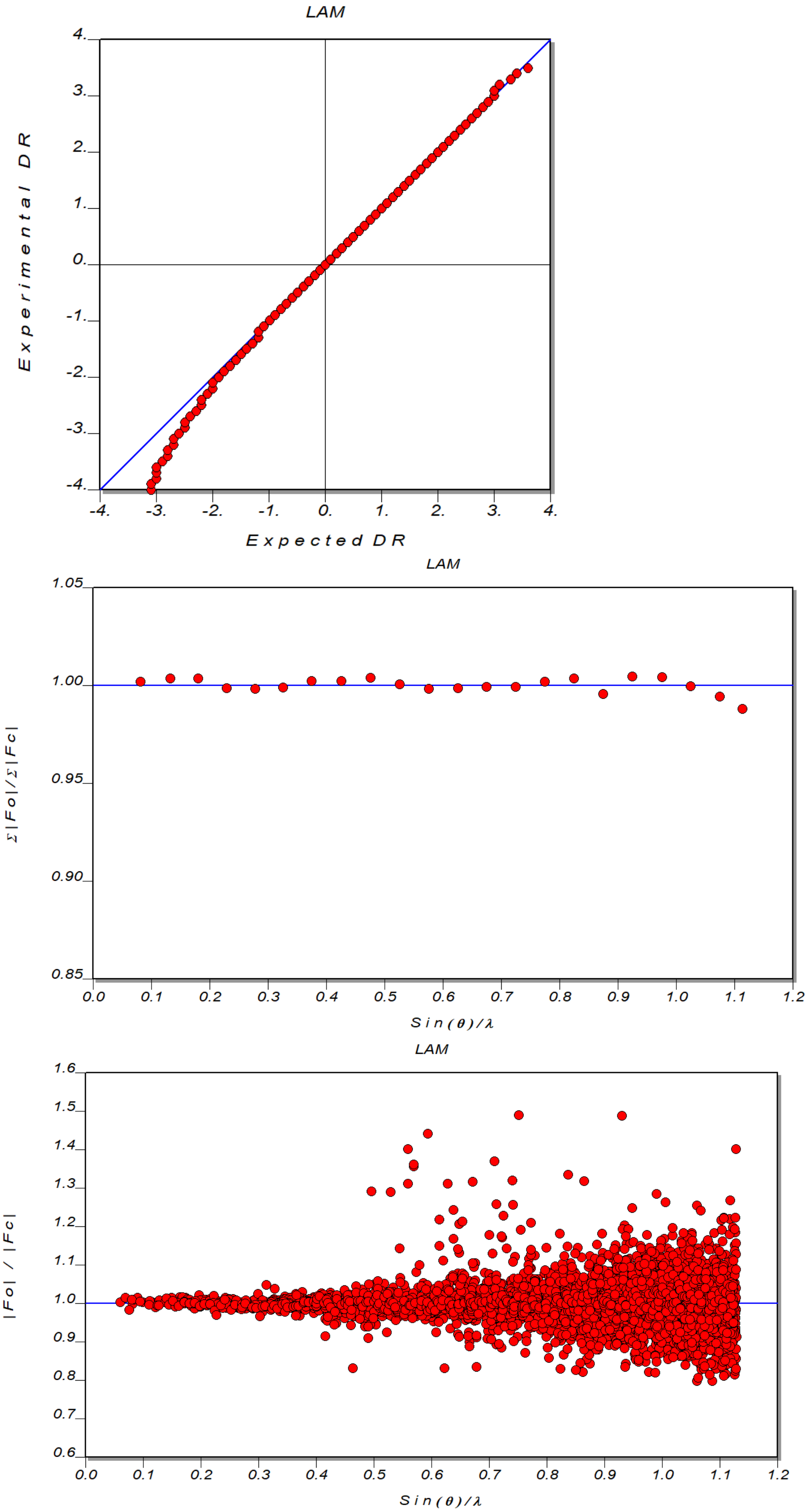

Figure S1. (a) Normal probability plot plotted against full dataset of *Lamivudine*; (b) Scale factor plot against resolution; (c) |Fo| / |Fc| against resolution.

Residual density map

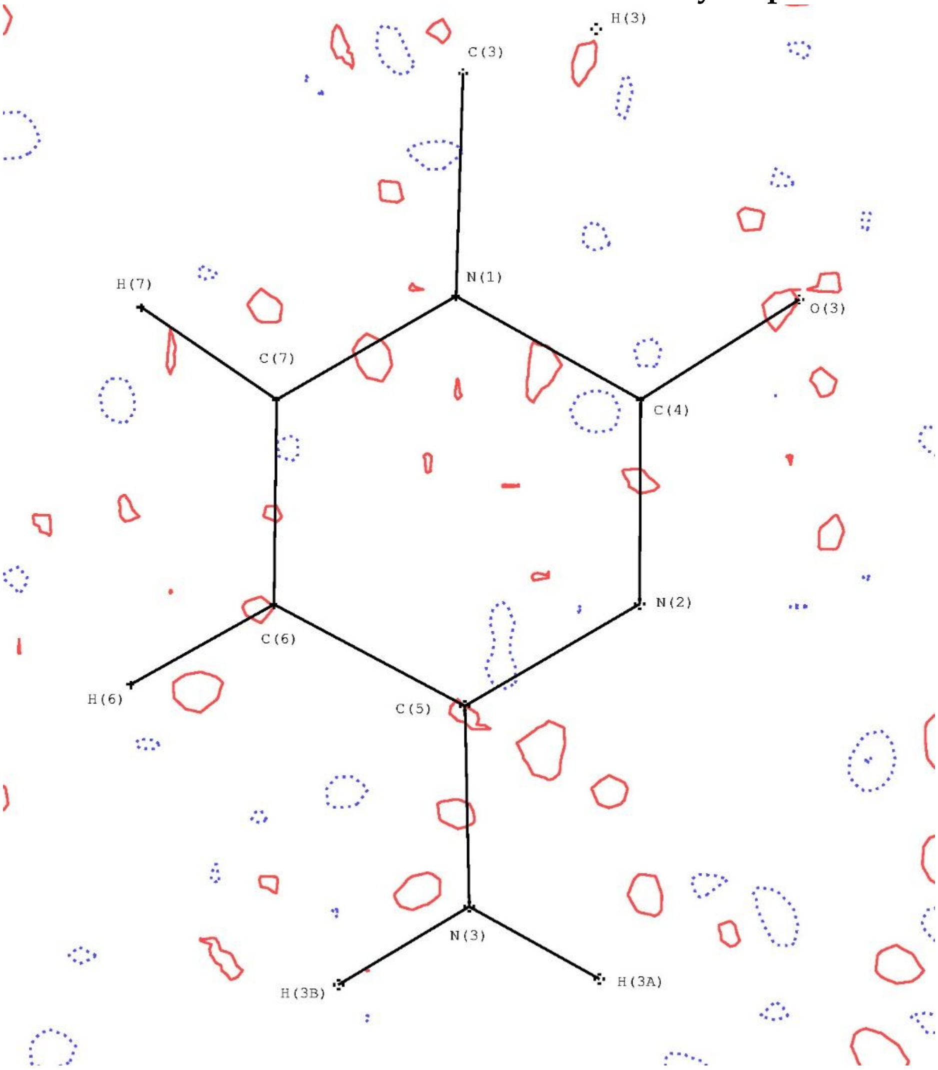

Figure S2. The residual density map in the section of (left) N(1), C(2) and C(4); (right) O(1), O(2) and C(24) atoms calculated using all data for *Lamivudine*. The isocontours are drawn every 0.05 eÅ-3; positive contours are shown in red, the negative contours are dashed blue.

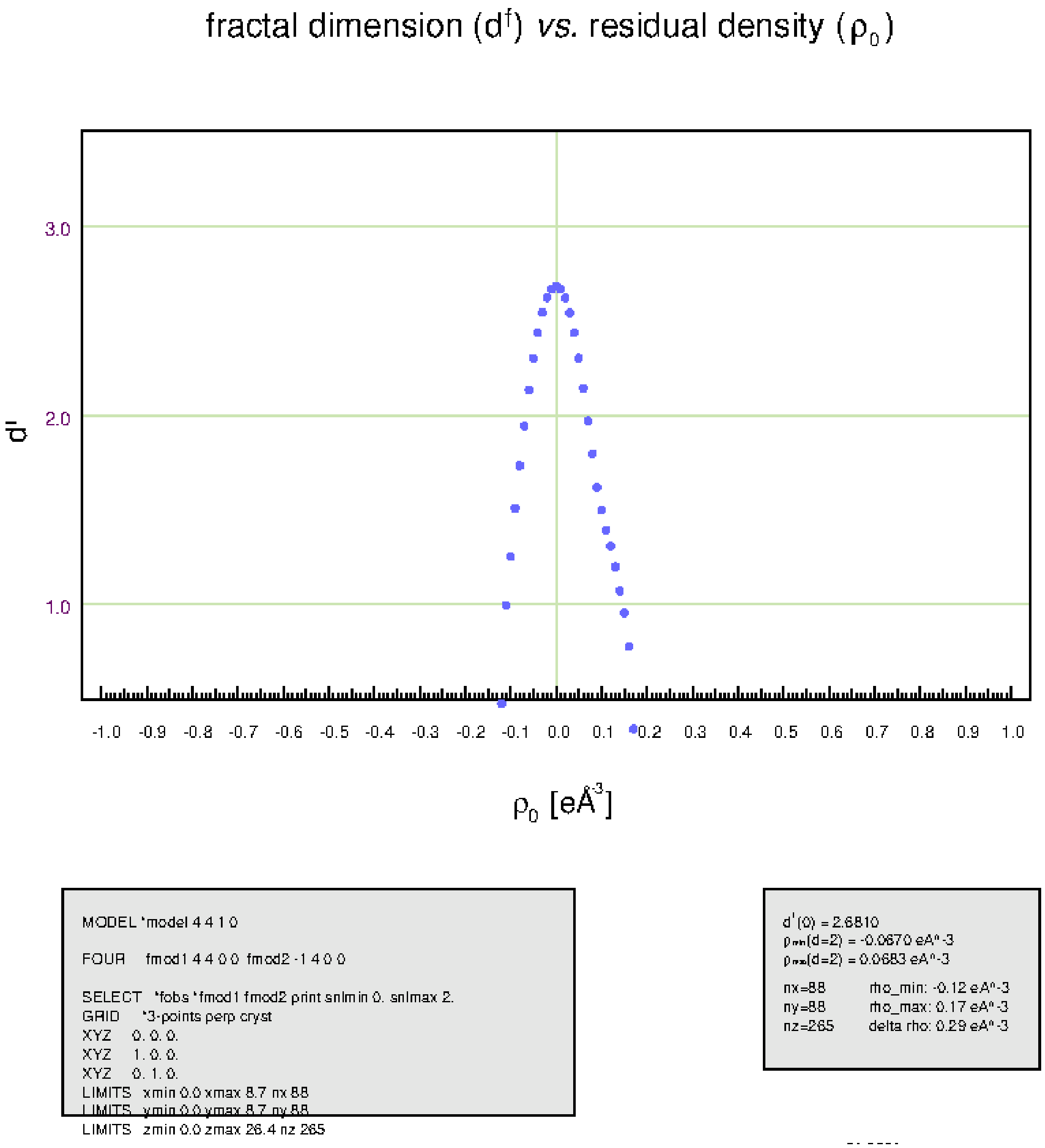

Figure S3. Plot of the fractal dimension df vs. the residual electron density (ϱ0) in the unit cell of *Lamivudine*. No resolution cutoff was applied to the data used for the Fourier transformation.

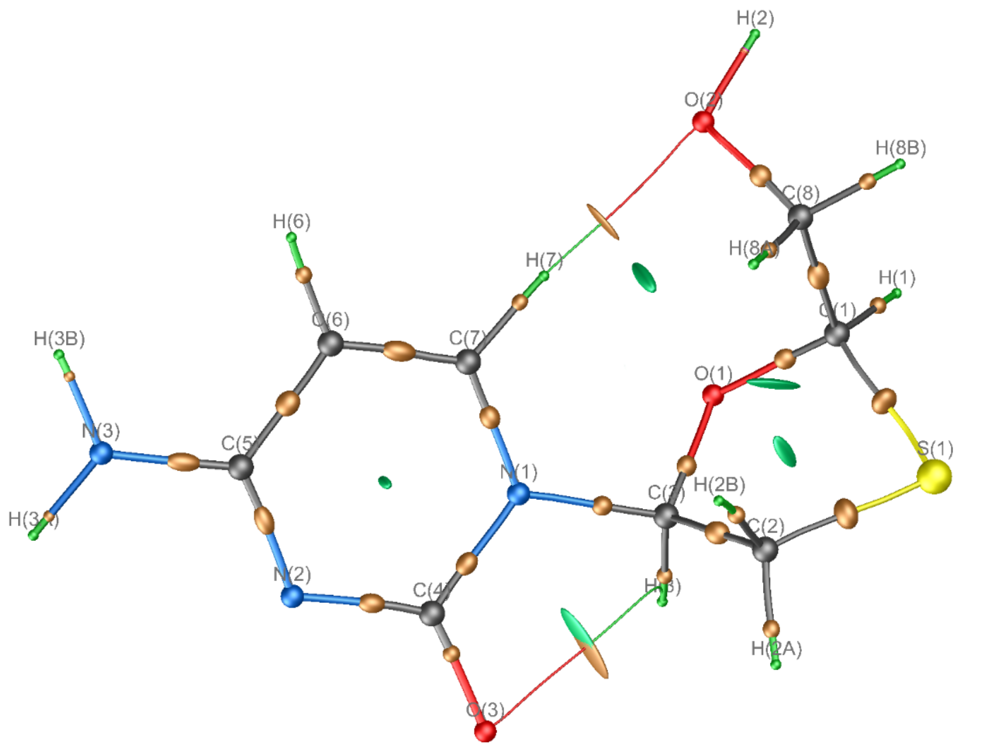

Figure S4. Molecular graph of *Lamivudine.* Orange and green ellipsoids denote bond and ring critical points.

Atomic coordinates of complexes A and B in pdb format.

```
Model1
REMARK   Generated by Multiwfn, Totally      379 atoms
HETATM    1  N   MOL A   1     -6.362   0.954  -7.061  1.00  0.00           N
HETATM    2  H   MOL A   1     -5.915   1.554  -7.746  1.00  0.00           H
HETATM    3  C   MOL A   1     -6.691   1.495  -5.732  1.00  0.00           C
HETATM    4  H   MOL A   1     -6.520   0.718  -4.987  1.00  0.00           H
HETATM    5  C   MOL A   1     -5.765   2.668  -5.349  1.00  0.00           C
HETATM    6  H   MOL A   1     -5.861   3.455  -6.093  1.00  0.00           H
HETATM    7  C   MOL A   1     -6.172   3.232  -3.979  1.00  0.00           C
HETATM    8  H   MOL A   1     -5.972   2.496  -3.201  1.00  0.00           H
HETATM    9  H   MOL A   1     -5.604   4.137  -3.764  1.00  0.00           H
HETATM   10  H   MOL A   1     -7.228   3.495  -3.959  1.00  0.00           H
HETATM   11  C   MOL A   1     -4.300   2.160  -5.339  1.00  0.00           C
HETATM   12  H   MOL A   1     -4.243   1.238  -4.759  1.00  0.00           H
HETATM   13  H   MOL A   1     -4.008   1.933  -6.363  1.00  0.00           H
HETATM   14  C   MOL A   1     -3.256   3.125  -4.771  1.00  0.00           C
HETATM   15  H   MOL A   1     -3.415   3.266  -3.703  1.00  0.00           H
HETATM   16  H   MOL A   1     -2.259   2.711  -4.922  1.00  0.00           H
HETATM   17  H   MOL A   1     -3.319   4.084  -5.275  1.00  0.00           H
HETATM   18  C   MOL A   1     -8.201   1.798  -5.731  1.00  0.00           C
HETATM   19  O   MOL A   1     -8.668   2.587  -6.547  1.00  0.00           O
HETATM   20  N   MOL A   1     -7.287  -3.804   4.074  1.00  0.00           N
HETATM   21  H   MOL A   1     -8.123  -3.610   3.521  1.00  0.00           H
HETATM   22  C   MOL A   1     -5.986  -3.760   3.371  1.00  0.00           C
HETATM   23  H   MOL A   1     -5.756  -4.791   3.112  1.00  0.00           H
HETATM   24  C   MOL A   1     -6.137  -2.975   2.049  1.00  0.00           C
HETATM   25  H   MOL A   1     -6.963  -3.394   1.479  1.00  0.00           H
HETATM   26  H   MOL A   1     -6.367  -1.933   2.271  1.00  0.00           H
HETATM   27  C   MOL A   1     -4.861  -3.052   1.195  1.00  0.00           C
HETATM   28  H   MOL A   1     -4.069  -2.485   1.686  1.00  0.00           H
HETATM   29  H   MOL A   1     -4.558  -4.098   1.143  1.00  0.00           H
HETATM   30  C   MOL A   1     -4.985  -2.558  -0.249  1.00  0.00           C
HETATM   31  O   MOL A   1     -4.091  -2.913  -1.042  1.00  0.00           O
HETATM   32  O   MOL A   1     -5.921  -1.792  -0.604  1.00  0.00           O
HETATM   33  C   MOL A   1     -4.827  -3.192   4.238  1.00  0.00           C
HETATM   34  O   MOL A   1     -5.054  -2.223   4.970  1.00  0.00           O
HETATM   35  N   MOL A   1     -2.495  -1.015   4.590  1.00  0.00           N
HETATM   36  H   MOL A   1     -3.199  -1.232   3.899  1.00  0.00           H
HETATM   37  C   MOL A   1     -1.978   0.365   4.666  1.00  0.00           C
HETATM   38  H   MOL A   1     -0.912   0.310   4.452  1.00  0.00           H
HETATM   39  C   MOL A   1     -2.606   1.248   3.570  1.00  0.00           C
HETATM   40  H   MOL A   1     -2.482   0.736   2.614  1.00  0.00           H
HETATM   41  C   MOL A   1     -4.103   1.499   3.781  1.00  0.00           C
HETATM   42  H   MOL A   1     -4.267   2.075   4.690  1.00  0.00           H
HETATM   43  H   MOL A   1     -4.494   2.063   2.933  1.00  0.00           H
HETATM   44  H   MOL A   1     -4.643   0.554   3.837  1.00  0.00           H
HETATM   45  C   MOL A   1     -1.878   2.592   3.478  1.00  0.00           C
HETATM   46  H   MOL A   1     -0.821   2.424   3.280  1.00  0.00           H
HETATM   47  H   MOL A   1     -2.299   3.180   2.663  1.00  0.00           H
HETATM   48  H   MOL A   1     -1.981   3.155   4.404  1.00  0.00           H
HETATM   49  C   MOL A   1     -2.085   0.990   6.066  1.00  0.00           C
HETATM   50  O   MOL A   1     -1.185   1.724   6.468  1.00  0.00           O
```

HETATM	51	N	MOL A	1	1.137	0.477	7.673	1.00	0.00	N
HETATM	52	H	MOL A	1	0.191	0.728	7.417	1.00	0.00	H
HETATM	53	C	MOL A	1	2.225	1.169	6.962	1.00	0.00	C
HETATM	54	H	MOL A	1	3.186	0.775	7.294	1.00	0.00	H
HETATM	55	C	MOL A	1	2.098	0.923	5.452	1.00	0.00	C
HETATM	56	H	MOL A	1	1.177	1.387	5.098	1.00	0.00	H
HETATM	57	H	MOL A	1	2.926	1.430	4.955	1.00	0.00	H
HETATM	58	C	MOL A	1	2.104	-0.504	4.996	1.00	0.00	C
HETATM	59	C	MOL A	1	2.691	-1.543	5.633	1.00	0.00	C
HETATM	60	H	MOL A	1	3.222	-1.485	6.574	1.00	0.00	H
HETATM	61	N	MOL A	1	2.518	-2.697	4.902	1.00	0.00	N
HETATM	62	H	MOL A	1	2.908	-3.589	5.195	1.00	0.00	H
HETATM	63	C	MOL A	1	1.837	-2.463	3.731	1.00	0.00	C
HETATM	64	C	MOL A	1	1.457	-3.288	2.665	1.00	0.00	C
HETATM	65	H	MOL A	1	1.725	-4.335	2.661	1.00	0.00	H
HETATM	66	C	MOL A	1	0.739	-2.727	1.597	1.00	0.00	C
HETATM	67	H	MOL A	1	0.453	-3.345	0.761	1.00	0.00	H
HETATM	68	C	MOL A	1	0.426	-1.355	1.605	1.00	0.00	C
HETATM	69	H	MOL A	1	-0.118	-0.920	0.778	1.00	0.00	H
HETATM	70	C	MOL A	1	0.846	-0.530	2.663	1.00	0.00	C
HETATM	71	H	MOL A	1	0.629	0.527	2.633	1.00	0.00	H
HETATM	72	C	MOL A	1	1.552	-1.064	3.763	1.00	0.00	C
HETATM	73	C	MOL A	1	2.236	2.673	7.252	1.00	0.00	C
HETATM	74	O	MOL A	1	3.312	3.244	7.426	1.00	0.00	O
HETATM	75	N	MOL A	1	5.253	4.018	4.256	1.00	0.00	N
HETATM	76	H	MOL A	1	4.608	3.942	5.030	1.00	0.00	H
HETATM	77	C	MOL A	1	4.730	4.423	2.947	1.00	0.00	C
HETATM	78	H	MOL A	1	5.162	3.774	2.182	1.00	0.00	H
HETATM	79	C	MOL A	1	3.201	4.231	2.953	1.00	0.00	C
HETATM	80	H	MOL A	1	2.988	3.164	3.032	1.00	0.00	H
HETATM	81	H	MOL A	1	2.790	4.723	3.836	1.00	0.00	H
HETATM	82	C	MOL A	1	2.478	4.796	1.716	1.00	0.00	C
HETATM	83	H	MOL A	1	2.635	5.872	1.667	1.00	0.00	H
HETATM	84	C	MOL A	1	2.964	4.156	0.417	1.00	0.00	C
HETATM	85	H	MOL A	1	2.819	3.079	0.451	1.00	0.00	H
HETATM	86	H	MOL A	1	2.411	4.569	-0.427	1.00	0.00	H
HETATM	87	H	MOL A	1	4.019	4.371	0.264	1.00	0.00	H
HETATM	88	C	MOL A	1	0.979	4.536	1.849	1.00	0.00	C
HETATM	89	H	MOL A	1	0.600	4.999	2.759	1.00	0.00	H
HETATM	90	H	MOL A	1	0.458	4.966	0.994	1.00	0.00	H
HETATM	91	H	MOL A	1	0.792	3.464	1.878	1.00	0.00	H
HETATM	92	C	MOL A	1	5.151	5.867	2.620	1.00	0.00	C
HETATM	93	O	MOL A	1	5.608	6.155	1.516	1.00	0.00	O
HETATM	94	N	MOL A	1	8.771	6.032	1.447	1.00	0.00	N
HETATM	95	H	MOL A	1	8.046	5.580	1.992	1.00	0.00	H
HETATM	96	C	MOL A	1	8.805	5.819	-0.004	1.00	0.00	C
HETATM	97	H	MOL A	1	9.832	5.595	-0.283	1.00	0.00	H
HETATM	98	C	MOL A	1	7.965	4.593	-0.392	1.00	0.00	C
HETATM	99	H	MOL A	1	8.336	3.743	0.181	1.00	0.00	H
HETATM	100	H	MOL A	1	6.921	4.744	-0.128	1.00	0.00	H
HETATM	101	C	MOL A	1	8.076	4.238	-1.884	1.00	0.00	C
HETATM	102	H	MOL A	1	9.119	4.334	-2.189	1.00	0.00	H
HETATM	103	H	MOL A	1	7.802	3.192	-2.007	1.00	0.00	H

```
HETATM 104 S MOL A 1 7.052 5.198 -3.038 1.00 0.00 S
HETATM 105 C MOL A 1 5.386 4.696 -2.526 1.00 0.00 C
HETATM 106 H MOL A 1 5.155 5.133 -1.555 1.00 0.00 H
HETATM 107 H MOL A 1 4.660 5.053 -3.257 1.00 0.00 H
HETATM 108 H MOL A 1 5.323 3.610 -2.461 1.00 0.00 H
HETATM 109 C MOL A 1 8.435 7.097 -0.770 1.00 0.00 C
HETATM 110 O MOL A 1 9.193 7.491 -1.650 1.00 0.00 O
HETATM 111 N MOL A 1 7.362 7.804 -0.398 1.00 0.00 N
HETATM 112 H MOL A 1 6.762 7.435 0.332 1.00 0.00 H
HETATM 113 C MOL A 1 7.022 9.099 -1.012 1.00 0.00 C
HETATM 114 H MOL A 1 6.894 8.954 -2.084 1.00 0.00 H
HETATM 115 C MOL A 1 5.708 9.639 -0.424 1.00 0.00 C
HETATM 116 H MOL A 1 5.871 9.847 0.634 1.00 0.00 H
HETATM 117 H MOL A 1 5.502 10.597 -0.903 1.00 0.00 H
HETATM 118 C MOL A 1 4.446 8.798 -0.556 1.00 0.00 C
HETATM 119 C MOL A 1 3.422 8.971 0.397 1.00 0.00 C
HETATM 120 H MOL A 1 3.571 9.648 1.227 1.00 0.00 H
HETATM 121 C MOL A 1 2.198 8.291 0.262 1.00 0.00 C
HETATM 122 H MOL A 1 1.417 8.423 0.995 1.00 0.00 H
HETATM 123 C MOL A 1 1.985 7.439 -0.843 1.00 0.00 C
HETATM 124 O MOL A 1 0.767 6.865 -1.023 1.00 0.00 O
HETATM 125 H MOL A 1 0.068 7.483 -0.767 1.00 0.00 H
HETATM 126 C MOL A 1 3.010 7.248 -1.790 1.00 0.00 C
HETATM 127 H MOL A 1 2.832 6.600 -2.636 1.00 0.00 H
HETATM 128 C MOL A 1 4.238 7.927 -1.648 1.00 0.00 C
HETATM 129 H MOL A 1 5.008 7.792 -2.395 1.00 0.00 H
HETATM 130 C MOL A 1 8.123 10.168 -0.840 1.00 0.00 C
HETATM 131 O MOL A 1 8.376 10.941 -1.760 1.00 0.00 O
HETATM 132 N MOL A 1 10.723 -0.334 1.845 1.00 0.00 N
HETATM 133 H MOL A 1 11.072 0.397 1.232 1.00 0.00 H
HETATM 134 C MOL A 1 9.276 -0.417 2.053 1.00 0.00 C
HETATM 135 H MOL A 1 9.111 -0.474 3.136 1.00 0.00 H
HETATM 136 C MOL A 1 8.636 0.894 1.546 1.00 0.00 C
HETATM 137 H MOL A 1 8.953 1.716 2.189 1.00 0.00 H
HETATM 138 H MOL A 1 9.029 1.101 0.548 1.00 0.00 H
HETATM 139 C MOL A 1 7.113 0.902 1.464 1.00 0.00 C
HETATM 140 C MOL A 1 6.325 0.458 2.545 1.00 0.00 C
HETATM 141 H MOL A 1 6.796 0.105 3.448 1.00 0.00 H
HETATM 142 C MOL A 1 4.924 0.406 2.435 1.00 0.00 C
HETATM 143 H MOL A 1 4.329 0.023 3.251 1.00 0.00 H
HETATM 144 C MOL A 1 4.299 0.807 1.244 1.00 0.00 C
HETATM 145 H MOL A 1 3.224 0.738 1.144 1.00 0.00 H
HETATM 146 C MOL A 1 5.076 1.274 0.171 1.00 0.00 C
HETATM 147 H MOL A 1 4.595 1.579 -0.748 1.00 0.00 H
HETATM 148 C MOL A 1 6.476 1.313 0.277 1.00 0.00 C
HETATM 149 H MOL A 1 7.062 1.638 -0.568 1.00 0.00 H
HETATM 150 C MOL A 1 8.771 -1.515 1.323 1.00 0.00 C
HETATM 151 O MOL A 1 8.027 -1.985 2.268 1.00 0.00 O
HETATM 152 N MOL A 1 8.903 -2.003 0.067 1.00 0.00 N
HETATM 153 H MOL A 1 9.695 -1.654 -0.485 1.00 0.00 H
HETATM 154 C MOL A 1 8.321 -3.306 -0.347 1.00 0.00 C
HETATM 155 H MOL A 1 7.230 -3.264 -0.014 1.00 0.00 H
HETATM 156 C MOL A 1 8.198 -3.409 -1.888 1.00 0.00 C
```

HETATM	157	H	MOL	A	1	9.202	-3.537	-2.327	1.00	0.00	H
HETATM	158	H	MOL	A	1	7.622	-4.327	-2.083	1.00	0.00	H
HETATM	159	C	MOL	A	1	7.505	-2.183	-2.494	1.00	0.00	C
HETATM	160	H	MOL	A	1	8.095	-1.264	-2.350	1.00	0.00	H
HETATM	161	H	MOL	A	1	7.412	-2.310	-3.584	1.00	0.00	H
HETATM	162	C	MOL	A	1	6.108	-1.942	-1.928	1.00	0.00	C
HETATM	163	O	MOL	A	1	5.598	-2.744	-1.097	1.00	0.00	O
HETATM	164	N	MOL	A	1	5.451	-0.858	-2.358	1.00	0.00	N
HETATM	165	H	MOL	A	1	4.454	-0.664	-2.059	1.00	0.00	H
HETATM	166	H	MOL	A	1	5.897	-0.260	-3.049	1.00	0.00	H
HETATM	167	C	MOL	A	1	8.696	-4.405	0.483	1.00	0.00	C
HETATM	168	O	MOL	A	1	7.860	-5.214	0.881	1.00	0.00	O
HETATM	169	N	MOL	A	1	6.800	-4.109	3.662	1.00	0.00	N
HETATM	170	H	MOL	A	1	7.378	-3.542	3.050	1.00	0.00	H
HETATM	171	C	MOL	A	1	5.388	-4.248	3.311	1.00	0.00	C
HETATM	172	H	MOL	A	1	4.795	-3.811	4.108	1.00	0.00	H
HETATM	173	C	MOL	A	1	5.089	-3.443	2.048	1.00	0.00	C
HETATM	174	H	MOL	A	1	5.627	-3.869	1.214	1.00	0.00	H
HETATM	175	H	MOL	A	1	4.025	-3.485	1.822	1.00	0.00	H
HETATM	176	H	MOL	A	1	5.379	-2.401	2.181	1.00	0.00	H
HETATM	177	C	MOL	A	1	4.960	-5.718	3.165	1.00	0.00	C
HETATM	178	O	MOL	A	1	3.862	-6.047	3.592	1.00	0.00	O
HETATM	179	N	MOL	A	1	2.299	-6.978	5.638	1.00	0.00	N
HETATM	180	H	MOL	A	1	3.152	-6.991	5.085	1.00	0.00	H
HETATM	181	C	MOL	A	1	1.083	-7.540	5.019	1.00	0.00	C
HETATM	182	H	MOL	A	1	0.250	-6.868	5.215	1.00	0.00	H
HETATM	183	C	MOL	A	1	1.233	-7.700	3.490	1.00	0.00	C
HETATM	184	H	MOL	A	1	1.271	-6.708	3.039	1.00	0.00	H
HETATM	185	H	MOL	A	1	2.163	-8.217	3.256	1.00	0.00	H
HETATM	186	C	MOL	A	1	0.064	-8.503	2.881	1.00	0.00	C
HETATM	187	H	MOL	A	1	0.095	-9.527	3.256	1.00	0.00	H
HETATM	188	H	MOL	A	1	-0.882	-8.068	3.192	1.00	0.00	H
HETATM	189	C	MOL	A	1	0.092	-8.605	1.357	1.00	0.00	C
HETATM	190	H	MOL	A	1	1.014	-9.109	1.072	1.00	0.00	H
HETATM	191	H	MOL	A	1	-0.748	-9.218	1.028	1.00	0.00	H
HETATM	192	N	MOL	A	1	0.065	-7.304	0.678	1.00	0.00	N
HETATM	193	H	MOL	A	1	0.971	-6.999	0.311	1.00	0.00	H
HETATM	194	C	MOL	A	1	-0.970	-6.545	0.388	1.00	0.00	C
HETATM	195	N	MOL	A	1	-2.171	-6.804	0.830	1.00	0.00	N
HETATM	196	H	MOL	A	1	-2.311	-7.601	1.432	1.00	0.00	H
HETATM	197	H	MOL	A	1	-2.954	-6.226	0.581	1.00	0.00	H
HETATM	198	N	MOL	A	1	-0.807	-5.495	-0.356	1.00	0.00	N
HETATM	199	H	MOL	A	1	0.129	-5.361	-0.758	1.00	0.00	H
HETATM	200	H	MOL	A	1	-1.568	-4.899	-0.612	1.00	0.00	H
HETATM	201	C	MOL	A	1	0.727	-8.868	5.661	1.00	0.00	C
HETATM	202	O	MOL	A	1	-0.426	-9.060	6.023	1.00	0.00	O
HETATM	203	N	MOL	A	1	-12.032	6.951	1.769	1.00	0.00	N
HETATM	204	H	MOL	A	1	-12.464	6.317	1.110	1.00	0.00	H
HETATM	205	C	MOL	A	1	-10.928	6.474	2.627	1.00	0.00	C
HETATM	206	H	MOL	A	1	-11.274	6.549	3.659	1.00	0.00	H
HETATM	207	C	MOL	A	1	-10.591	4.998	2.359	1.00	0.00	C
HETATM	208	H	MOL	A	1	-11.458	4.383	2.606	1.00	0.00	H
HETATM	209	H	MOL	A	1	-10.327	4.852	1.311	1.00	0.00	H

HETATM	210	C	MOL	A	1	-9.401	4.607	3.247	1.00	0.00	C
HETATM	211	H	MOL	A	1	-8.491	5.034	2.823	1.00	0.00	H
HETATM	212	H	MOL	A	1	-9.555	5.028	4.243	1.00	0.00	H
HETATM	213	C	MOL	A	1	-9.176	3.111	3.417	1.00	0.00	C
HETATM	214	H	MOL	A	1	-10.102	2.654	3.768	1.00	0.00	H
HETATM	215	H	MOL	A	1	-8.879	2.678	2.460	1.00	0.00	H
HETATM	216	N	MOL	A	1	-8.116	2.906	4.412	1.00	0.00	N
HETATM	217	H	MOL	A	1	-7.588	3.739	4.682	1.00	0.00	H
HETATM	218	C	MOL	A	1	-7.788	1.794	5.033	1.00	0.00	C
HETATM	219	N	MOL	A	1	-8.382	0.656	4.828	1.00	0.00	N
HETATM	220	H	MOL	A	1	-9.125	0.594	4.144	1.00	0.00	H
HETATM	221	H	MOL	A	1	-8.031	-0.150	5.325	1.00	0.00	H
HETATM	222	N	MOL	A	1	-6.839	1.790	5.911	1.00	0.00	N
HETATM	223	H	MOL	A	1	-6.306	2.652	6.058	1.00	0.00	H
HETATM	224	H	MOL	A	1	-6.595	0.918	6.357	1.00	0.00	H
HETATM	225	C	MOL	A	1	-9.690	7.371	2.503	1.00	0.00	C
HETATM	226	O	MOL	A	1	-9.100	7.457	1.425	1.00	0.00	O
HETATM	227	N	MOL	A	1	-6.121	6.688	0.739	1.00	0.00	N
HETATM	228	H	MOL	A	1	-6.976	6.800	1.266	1.00	0.00	H
HETATM	229	C	MOL	A	1	-6.226	6.494	-0.716	1.00	0.00	C
HETATM	230	H	MOL	A	1	-5.279	6.114	-1.100	1.00	0.00	H
HETATM	231	C	MOL	A	1	-7.298	5.430	-1.039	1.00	0.00	C
HETATM	232	H	MOL	A	1	-8.237	5.711	-0.560	1.00	0.00	H
HETATM	233	H	MOL	A	1	-7.456	5.411	-2.117	1.00	0.00	H
HETATM	234	C	MOL	A	1	-6.887	4.009	-0.596	1.00	0.00	C
HETATM	235	H	MOL	A	1	-5.943	3.755	-1.080	1.00	0.00	H
HETATM	236	H	MOL	A	1	-6.732	4.005	0.485	1.00	0.00	H
HETATM	237	C	MOL	A	1	-7.930	2.929	-0.935	1.00	0.00	C
HETATM	238	O	MOL	A	1	-9.148	3.221	-0.891	1.00	0.00	O
HETATM	239	O	MOL	A	1	-7.572	1.740	-1.154	1.00	0.00	O
HETATM	240	C	MOL	A	1	-6.480	7.825	-1.464	1.00	0.00	C
HETATM	241	O	MOL	A	1	-6.151	7.922	-2.646	1.00	0.00	O
HETATM	242	O	MOL	A	1	2.544	1.608	-2.298	1.00	0.00	O
HETATM	243	C	MOL	A	1	1.991	0.510	-2.053	1.00	0.00	C
HETATM	244	N	MOL	A	1	2.687	-0.613	-1.738	1.00	0.00	N
HETATM	245	C	MOL	A	1	2.062	-1.808	-1.567	1.00	0.00	C
HETATM	246	N	MOL	A	1	2.791	-2.877	-1.257	1.00	0.00	N
HETATM	247	H	MOL	A	1	2.404	-3.850	-1.270	1.00	0.00	H
HETATM	248	H	MOL	A	1	3.808	-2.754	-1.126	1.00	0.00	H
HETATM	249	C	MOL	A	1	0.631	-1.923	-1.709	1.00	0.00	C
HETATM	250	H	MOL	A	1	0.137	-2.893	-1.674	1.00	0.00	H
HETATM	251	C	MOL	A	1	-0.066	-0.776	-1.943	1.00	0.00	C
HETATM	252	H	MOL	A	1	-1.146	-0.753	-2.101	1.00	0.00	H
HETATM	253	N	MOL	A	1	0.563	0.438	-2.052	1.00	0.00	N
HETATM	254	C	MOL	A	1	-0.187	1.693	-2.353	1.00	0.00	C
HETATM	255	O	MOL	A	1	-1.576	1.427	-2.492	1.00	0.00	O
HETATM	256	H	MOL	A	1	0.191	2.107	-3.303	1.00	0.00	H
HETATM	257	C	MOL	A	1	0.000	2.710	-1.227	1.00	0.00	C
HETATM	258	H	MOL	A	1	1.029	2.684	-0.855	1.00	0.00	H
HETATM	259	H	MOL	A	1	-0.242	3.724	-1.583	1.00	0.00	H
HETATM	260	S	MOL	A	1	-1.179	2.143	0.045	1.00	0.00	S
HETATM	261	C	MOL	A	1	-2.359	1.795	-1.343	1.00	0.00	C
HETATM	262	H	MOL	A	1	-2.925	2.721	-1.558	1.00	0.00	H

HETATM 263 C MOL A 1 -3.344 0.708 -0.945 1.00 0.00 C
HETATM 264 H MOL A 1 -3.715 0.908 0.071 1.00 0.00 H
HETATM 265 H MOL A 1 -2.851 -0.281 -0.956 1.00 0.00 H
HETATM 266 O MOL A 1 -4.492 0.700 -1.846 1.00 0.00 O
HETATM 267 H MOL A 1 -5.261 0.694 -1.221 1.00 0.00 H
HETATM 268 C MOL A 1 -6.359 -6.903 0.247 1.00 0.00 C
HETATM 269 O MOL A 1 -5.611 -6.598 1.260 1.00 0.00 O
HETATM 270 N MOL A 1 -6.020 -6.763 -1.088 1.00 0.00 N
HETATM 271 H MOL A 1 -6.728 -7.228 -1.682 1.00 0.00 H
HETATM 272 C MOL A 1 -4.606 -7.247 -1.575 1.00 0.00 C
HETATM 273 H MOL A 1 -4.875 -7.878 -2.507 1.00 0.00 H
HETATM 274 C MOL A 1 -3.737 -6.052 -1.961 1.00 0.00 C
HETATM 275 H MOL A 1 -3.667 -5.430 -1.056 1.00 0.00 H
HETATM 276 H MOL A 1 -2.722 -6.404 -2.208 1.00 0.00 H
HETATM 277 C MOL A 1 -4.311 -5.251 -3.133 1.00 0.00 C
HETATM 278 H MOL A 1 -4.708 -5.936 -3.900 1.00 0.00 H
HETATM 279 H MOL A 1 -5.171 -4.640 -2.802 1.00 0.00 H
HETATM 280 C MOL A 1 -3.224 -4.381 -3.777 1.00 0.00 C
HETATM 281 H MOL A 1 -2.725 -3.735 -3.037 1.00 0.00 H
HETATM 282 H MOL A 1 -2.413 -4.990 -4.220 1.00 0.00 H
HETATM 283 N MOL A 1 -3.793 -3.519 -4.825 1.00 0.00 N
HETATM 284 H MOL A 1 -4.112 -4.015 -5.668 1.00 0.00 H
HETATM 285 C MOL A 1 -4.275 -2.284 -4.621 1.00 0.00 C
HETATM 286 N MOL A 1 -4.169 -1.676 -3.431 1.00 0.00 N
HETATM 287 H MOL A 1 -4.095 -2.274 -2.585 1.00 0.00 H
HETATM 288 H MOL A 1 -4.427 -0.713 -3.158 1.00 0.00 H
HETATM 289 N MOL A 1 -4.730 -1.559 -5.680 1.00 0.00 N
HETATM 290 H MOL A 1 -4.843 -2.104 -6.552 1.00 0.00 H
HETATM 291 H MOL A 1 -5.542 -0.972 -5.504 1.00 0.00 H
HETATM 292 C MOL A 1 -4.166 -8.503 -1.157 1.00 0.00 C
HETATM 293 O MOL A 1 -4.634 -9.058 -0.162 1.00 0.00 O
HETATM 294 C MOL A 1 0.373 -6.945 -4.479 1.00 0.00 C
HETATM 295 O MOL A 1 -0.089 -5.729 -4.413 1.00 0.00 O
HETATM 296 N MOL A 1 1.468 -7.368 -3.700 1.00 0.00 N
HETATM 297 H MOL A 1 1.699 -8.352 -3.893 1.00 0.00 H
HETATM 298 C MOL A 1 2.700 -6.510 -3.692 1.00 0.00 C
HETATM 299 H MOL A 1 2.333 -5.461 -3.655 1.00 0.00 H
HETATM 300 C MOL A 1 3.497 -6.781 -2.380 1.00 0.00 C
HETATM 301 H MOL A 1 3.756 -7.840 -2.269 1.00 0.00 H
HETATM 302 H MOL A 1 4.430 -6.197 -2.322 1.00 0.00 H
HETATM 303 C MOL A 1 2.573 -6.398 -1.127 1.00 0.00 C
HETATM 304 O MOL A 1 1.793 -5.397 -1.213 1.00 0.00 O
HETATM 305 O MOL A 1 2.732 -7.168 -0.161 1.00 0.00 O
HETATM 306 C MOL A 1 3.540 -6.920 -4.771 1.00 0.00 C
HETATM 307 O MOL A 1 3.853 -6.073 -5.606 1.00 0.00 O
HETATM 308 N MOL A 1 3.053 -3.516 -6.910 1.00 0.00 N
HETATM 309 H MOL A 1 2.336 -3.942 -6.343 1.00 0.00 H
HETATM 310 C MOL A 1 3.802 -2.391 -6.344 1.00 0.00 C
HETATM 311 H MOL A 1 3.707 -1.535 -7.014 1.00 0.00 H
HETATM 312 C MOL A 1 3.206 -2.002 -4.992 1.00 0.00 C
HETATM 313 H MOL A 1 3.248 -2.859 -4.317 1.00 0.00 H
HETATM 314 H MOL A 1 3.822 -1.215 -4.557 1.00 0.00 H
HETATM 315 C MOL A 1 1.786 -1.489 -5.071 1.00 0.00 C

HETATM	316	C	MOL	A	1	0.708	-2.358	-4.832	1.00	0.00	C
HETATM	317	H	MOL	A	1	0.889	-3.397	-4.599	1.00	0.00	H
HETATM	318	C	MOL	A	1	-0.603	-1.859	-4.823	1.00	0.00	C
HETATM	319	H	MOL	A	1	-1.412	-2.523	-4.579	1.00	0.00	H
HETATM	320	C	MOL	A	1	-0.844	-0.493	-5.048	1.00	0.00	C
HETATM	321	H	MOL	A	1	-1.848	-0.097	-4.976	1.00	0.00	H
HETATM	322	C	MOL	A	1	0.229	0.366	-5.337	1.00	0.00	C
HETATM	323	H	MOL	A	1	0.052	1.419	-5.512	1.00	0.00	H
HETATM	324	C	MOL	A	1	1.544	-0.130	-5.345	1.00	0.00	C
HETATM	325	H	MOL	A	1	2.370	0.543	-5.530	1.00	0.00	H
HETATM	326	C	MOL	A	1	5.298	-2.669	-6.193	1.00	0.00	C
HETATM	327	O	MOL	A	1	6.093	-1.758	-6.384	1.00	0.00	O
HETATM	328	O	MOL	A	1	1.655	3.560	-4.141	1.00	0.00	O
HETATM	329	H	MOL	A	1	2.207	3.647	-4.949	1.00	0.00	H
HETATM	330	H	MOL	A	1	2.146	2.914	-3.581	1.00	0.00	H
HETATM	331	O	MOL	A	1	-3.674	4.093	0.465	1.00	0.00	O
HETATM	332	H	MOL	A	1	-3.691	5.079	0.480	1.00	0.00	H
HETATM	333	H	MOL	A	1	-2.717	3.888	0.512	1.00	0.00	H
HETATM	334	O	MOL	A	1	-5.705	0.313	0.864	1.00	0.00	O
HETATM	335	H	MOL	A	1	-6.674	0.513	0.881	1.00	0.00	H
HETATM	336	H	MOL	A	1	-5.741	-0.508	0.271	1.00	0.00	H
HETATM	337	O	MOL	A	1	-10.965	-0.650	-0.813	1.00	0.00	O
HETATM	338	H	MOL	A	1	-10.323	0.087	-0.727	1.00	0.00	H
HETATM	339	H	MOL	A	1	-10.591	-1.253	-0.130	1.00	0.00	H
HETATM	340	O	MOL	A	1	-8.556	0.419	0.772	1.00	0.00	O
HETATM	341	H	MOL	A	1	-8.198	0.975	-0.017	1.00	0.00	H
HETATM	342	H	MOL	A	1	-8.413	-0.484	0.331	1.00	0.00	H
HETATM	343	O	MOL	A	1	5.841	-5.688	-0.631	1.00	0.00	O
HETATM	344	H	MOL	A	1	5.696	-4.739	-0.849	1.00	0.00	H
HETATM	345	H	MOL	A	1	6.707	-5.583	-0.106	1.00	0.00	H
HETATM	346	O	MOL	A	1	-8.471	-1.913	-0.648	1.00	0.00	O
HETATM	347	H	MOL	A	1	-8.379	-1.380	-1.478	1.00	0.00	H
HETATM	348	H	MOL	A	1	-7.475	-2.074	-0.512	1.00	0.00	H
HETATM	349	O	MOL	A	1	-7.329	-0.326	-2.697	1.00	0.00	O
HETATM	350	H	MOL	A	1	-7.388	0.512	-2.124	1.00	0.00	H
HETATM	351	H	MOL	A	1	-6.598	-0.767	-2.168	1.00	0.00	H
HETATM	352	H	MOL	A	1	8.671	10.235	0.088	1.00	0.00	H
HETATM	353	H	MOL	A	1	9.452	6.614	1.891	1.00	0.00	H
HETATM	354	H	MOL	A	1	5.043	6.637	3.370	1.00	0.00	H
HETATM	355	H	MOL	A	1	6.226	3.821	4.380	1.00	0.00	H
HETATM	356	H	MOL	A	1	11.346	-0.976	2.292	1.00	0.00	H
HETATM	357	H	MOL	A	1	9.731	-4.528	0.766	1.00	0.00	H
HETATM	358	H	MOL	A	1	-2.942	0.786	6.690	1.00	0.00	H
HETATM	359	H	MOL	A	1	-2.155	-1.722	5.210	1.00	0.00	H
HETATM	360	H	MOL	A	1	1.325	-0.208	8.377	1.00	0.00	H
HETATM	361	H	MOL	A	1	1.310	3.226	7.303	1.00	0.00	H
HETATM	362	H	MOL	A	1	-12.350	7.896	1.840	1.00	0.00	H
HETATM	363	H	MOL	A	1	-9.335	7.925	3.360	1.00	0.00	H
HETATM	364	H	MOL	A	1	-6.940	8.659	-0.954	1.00	0.00	H
HETATM	365	H	MOL	A	1	-5.229	6.708	1.192	1.00	0.00	H
HETATM	366	H	MOL	A	1	-8.848	1.316	-5.014	1.00	0.00	H
HETATM	367	H	MOL	A	1	-6.577	0.003	-7.286	1.00	0.00	H
HETATM	368	H	MOL	A	1	-3.393	-9.003	-1.721	1.00	0.00	H

```
HETATM 369 H MOL A 1 -7.339 -7.303 0.465 1.00 0.00 H
HETATM 370 H MOL A 1 -0.096 -7.649 -5.151 1.00 0.00 H
HETATM 371 H MOL A 1 3.890 -7.938 -4.850 1.00 0.00 H
HETATM 372 H MOL A 1 3.247 -3.853 -7.831 1.00 0.00 H
HETATM 373 H MOL A 1 5.648 -3.654 -5.922 1.00 0.00 H
HETATM 374 H MOL A 1 5.612 -6.441 2.699 1.00 0.00 H
HETATM 375 H MOL A 1 7.181 -4.555 4.472 1.00 0.00 H
HETATM 376 H MOL A 1 2.284 -6.599 6.563 1.00 0.00 H
HETATM 377 H MOL A 1 1.479 -9.631 5.797 1.00 0.00 H
HETATM 378 H MOL A 1 -7.345 -4.016 5.049 1.00 0.00 H
HETATM 379 H MOL A 1 -3.843 -3.635 4.201 1.00 0.00 H
END

Model2
REMARK Generated by Multiwfn, Totally 336 atoms
HETATM 1 N MOL A 1 -8.760 2.304 -0.217 1.00 0.00 N
HETATM 2 H MOL A 1 -7.892 2.741 -0.504 1.00 0.00 H
HETATM 3 H MOL A 1 -9.425 3.039 0.017 1.00 0.00 H
HETATM 4 H MOL A 1 -9.149 1.798 -1.006 1.00 0.00 H
HETATM 5 C MOL A 1 -8.558 1.362 0.907 1.00 0.00 C
HETATM 6 H MOL A 1 -9.507 0.849 1.073 1.00 0.00 H
HETATM 7 C MOL A 1 -7.502 0.293 0.559 1.00 0.00 C
HETATM 8 H MOL A 1 -7.529 -0.503 1.299 1.00 0.00 H
HETATM 9 H MOL A 1 -7.800 -0.169 -0.375 1.00 0.00 H
HETATM 10 C MOL A 1 -6.052 0.787 0.431 1.00 0.00 C
HETATM 11 H MOL A 1 -6.038 1.873 0.362 1.00 0.00 H
HETATM 12 H MOL A 1 -5.493 0.522 1.328 1.00 0.00 H
HETATM 13 C MOL A 1 -5.392 0.198 -0.821 1.00 0.00 C
HETATM 14 O MOL A 1 -4.360 -0.492 -0.700 1.00 0.00 O
HETATM 15 O MOL A 1 -5.945 0.411 -1.926 1.00 0.00 O
HETATM 16 C MOL A 1 -8.193 2.110 2.189 1.00 0.00 C
HETATM 17 O MOL A 1 -7.921 3.304 2.111 1.00 0.00 O
HETATM 18 N MOL A 1 -5.637 3.005 3.631 1.00 0.00 N
HETATM 19 H MOL A 1 -6.236 3.169 2.830 1.00 0.00 H
HETATM 20 C MOL A 1 -4.236 3.449 3.512 1.00 0.00 C
HETATM 21 H MOL A 1 -3.598 2.608 3.789 1.00 0.00 H
HETATM 22 C MOL A 1 -3.909 3.792 2.043 1.00 0.00 C
HETATM 23 H MOL A 1 -4.115 2.909 1.438 1.00 0.00 H
HETATM 24 C MOL A 1 -4.739 4.951 1.483 1.00 0.00 C
HETATM 25 H MOL A 1 -4.561 5.850 2.069 1.00 0.00 H
HETATM 26 H MOL A 1 -4.447 5.148 0.452 1.00 0.00 H
HETATM 27 H MOL A 1 -5.800 4.713 1.501 1.00 0.00 H
HETATM 28 C MOL A 1 -2.432 4.130 1.875 1.00 0.00 C
HETATM 29 H MOL A 1 -1.829 3.310 2.256 1.00 0.00 H
HETATM 30 H MOL A 1 -2.187 4.287 0.828 1.00 0.00 H
HETATM 31 H MOL A 1 -2.181 5.043 2.413 1.00 0.00 H
HETATM 32 C MOL A 1 -3.869 4.591 4.472 1.00 0.00 C
HETATM 33 O MOL A 1 -2.714 4.696 4.866 1.00 0.00 O
HETATM 34 N MOL A 1 -1.336 3.698 7.046 1.00 0.00 N
HETATM 35 H MOL A 1 -1.972 4.211 6.447 1.00 0.00 H
HETATM 36 C MOL A 1 0.015 3.471 6.515 1.00 0.00 C
HETATM 37 H MOL A 1 0.586 2.870 7.216 1.00 0.00 H
HETATM 38 C MOL A 1 -0.051 2.730 5.168 1.00 0.00 C
```

HETATM	39	H	MOL	A	1	-0.490	3.401	4.429	1.00	0.00	H
HETATM	40	H	MOL	A	1	0.975	2.551	4.853	1.00	0.00	H
HETATM	41	C	MOL	A	1	-0.759	1.414	5.043	1.00	0.00	C
HETATM	42	C	MOL	A	1	-1.279	0.653	6.032	1.00	0.00	C
HETATM	43	H	MOL	A	1	-1.265	0.879	7.086	1.00	0.00	H
HETATM	44	N	MOL	A	1	-1.801	-0.505	5.504	1.00	0.00	N
HETATM	45	H	MOL	A	1	-2.220	-1.226	6.085	1.00	0.00	H
HETATM	46	C	MOL	A	1	-1.558	-0.609	4.159	1.00	0.00	C
HETATM	47	C	MOL	A	1	-1.769	-1.627	3.223	1.00	0.00	C
HETATM	48	H	MOL	A	1	-2.224	-2.558	3.521	1.00	0.00	H
HETATM	49	C	MOL	A	1	-1.307	-1.446	1.911	1.00	0.00	C
HETATM	50	H	MOL	A	1	-1.373	-2.251	1.196	1.00	0.00	H
HETATM	51	C	MOL	A	1	-0.727	-0.227	1.535	1.00	0.00	C
HETATM	52	H	MOL	A	1	-0.377	-0.084	0.524	1.00	0.00	H
HETATM	53	C	MOL	A	1	-0.563	0.802	2.475	1.00	0.00	C
HETATM	54	H	MOL	A	1	-0.102	1.723	2.163	1.00	0.00	H
HETATM	55	C	MOL	A	1	-0.930	0.625	3.825	1.00	0.00	C
HETATM	56	C	MOL	A	1	0.824	4.753	6.279	1.00	0.00	C
HETATM	57	O	MOL	A	1	2.047	4.689	6.434	1.00	0.00	O
HETATM	58	N	MOL	A	1	4.389	4.826	4.820	1.00	0.00	N
HETATM	59	H	MOL	A	1	3.507	4.931	5.312	1.00	0.00	H
HETATM	60	C	MOL	A	1	4.257	4.726	3.359	1.00	0.00	C
HETATM	61	H	MOL	A	1	4.701	3.794	3.008	1.00	0.00	H
HETATM	62	C	MOL	A	1	2.748	4.687	3.100	1.00	0.00	C
HETATM	63	H	MOL	A	1	2.376	3.719	3.423	1.00	0.00	H
HETATM	64	H	MOL	A	1	2.289	5.437	3.739	1.00	0.00	H
HETATM	65	C	MOL	A	1	2.234	4.974	1.683	1.00	0.00	C
HETATM	66	H	MOL	A	1	2.481	5.996	1.399	1.00	0.00	H
HETATM	67	C	MOL	A	1	2.731	4.012	0.610	1.00	0.00	C
HETATM	68	H	MOL	A	1	2.504	2.982	0.878	1.00	0.00	H
HETATM	69	H	MOL	A	1	2.244	4.254	-0.335	1.00	0.00	H
HETATM	70	H	MOL	A	1	3.798	4.129	0.464	1.00	0.00	H
HETATM	71	C	MOL	A	1	0.725	4.833	1.776	1.00	0.00	C
HETATM	72	H	MOL	A	1	0.329	5.522	2.521	1.00	0.00	H
HETATM	73	H	MOL	A	1	0.277	5.051	0.810	1.00	0.00	H
HETATM	74	H	MOL	A	1	0.465	3.819	2.054	1.00	0.00	H
HETATM	75	C	MOL	A	1	4.931	5.879	2.596	1.00	0.00	C
HETATM	76	O	MOL	A	1	5.484	5.653	1.523	1.00	0.00	O
HETATM	77	N	MOL	A	1	8.115	4.367	1.530	1.00	0.00	N
HETATM	78	H	MOL	A	1	7.524	5.096	1.135	1.00	0.00	H
HETATM	79	C	MOL	A	1	7.785	2.973	1.206	1.00	0.00	C
HETATM	80	H	MOL	A	1	7.551	2.449	2.134	1.00	0.00	H
HETATM	81	C	MOL	A	1	6.524	2.941	0.330	1.00	0.00	C
HETATM	82	H	MOL	A	1	5.671	3.097	0.985	1.00	0.00	H
HETATM	83	H	MOL	A	1	6.563	3.747	-0.404	1.00	0.00	H
HETATM	84	C	MOL	A	1	6.312	1.618	-0.414	1.00	0.00	C
HETATM	85	H	MOL	A	1	7.017	1.553	-1.244	1.00	0.00	H
HETATM	86	H	MOL	A	1	6.516	0.796	0.271	1.00	0.00	H
HETATM	87	S	MOL	A	1	4.641	1.392	-1.060	1.00	0.00	S
HETATM	88	C	MOL	A	1	4.587	2.692	-2.321	1.00	0.00	C
HETATM	89	H	MOL	A	1	5.391	2.534	-3.036	1.00	0.00	H
HETATM	90	H	MOL	A	1	3.636	2.649	-2.847	1.00	0.00	H
HETATM	91	H	MOL	A	1	4.694	3.673	-1.860	1.00	0.00	H

HETATM	92	C	MOL	A	1	8.930	2.204	0.542	1.00	0.00	C
HETATM	93	O	MOL	A	1	9.224	1.092	0.979	1.00	0.00	O
HETATM	94	N	MOL	A	1	9.569	2.781	-0.477	1.00	0.00	N
HETATM	95	H	MOL	A	1	9.262	3.704	-0.750	1.00	0.00	H
HETATM	96	C	MOL	A	1	10.501	2.100	-1.392	1.00	0.00	C
HETATM	97	H	MOL	A	1	9.989	1.217	-1.774	1.00	0.00	H
HETATM	98	C	MOL	A	1	10.776	3.012	-2.596	1.00	0.00	C
HETATM	99	H	MOL	A	1	11.393	3.852	-2.272	1.00	0.00	H
HETATM	100	H	MOL	A	1	11.341	2.453	-3.343	1.00	0.00	H
HETATM	101	C	MOL	A	1	9.501	3.539	-3.228	1.00	0.00	C
HETATM	102	C	MOL	A	1	9.007	4.801	-2.851	1.00	0.00	C
HETATM	103	H	MOL	A	1	9.564	5.422	-2.163	1.00	0.00	H
HETATM	104	C	MOL	A	1	7.768	5.250	-3.340	1.00	0.00	C
HETATM	105	H	MOL	A	1	7.393	6.214	-3.034	1.00	0.00	H
HETATM	106	C	MOL	A	1	7.009	4.438	-4.209	1.00	0.00	C
HETATM	107	O	MOL	A	1	5.778	4.839	-4.623	1.00	0.00	O
HETATM	108	H	MOL	A	1	5.505	5.652	-4.146	1.00	0.00	H
HETATM	109	C	MOL	A	1	7.514	3.180	-4.604	1.00	0.00	C
HETATM	110	H	MOL	A	1	6.925	2.564	-5.266	1.00	0.00	H
HETATM	111	C	MOL	A	1	8.755	2.729	-4.108	1.00	0.00	C
HETATM	112	H	MOL	A	1	9.125	1.751	-4.380	1.00	0.00	H
HETATM	113	C	MOL	A	1	11.785	1.591	-0.731	1.00	0.00	C
HETATM	114	O	MOL	A	1	12.320	2.271	0.173	1.00	0.00	O
HETATM	115	O	MOL	A	1	12.143	0.423	-1.002	1.00	0.00	O
HETATM	116	N	MOL	A	1	7.961	-2.744	4.090	1.00	0.00	N
HETATM	117	H	MOL	A	1	8.691	-2.571	3.405	1.00	0.00	H
HETATM	118	C	MOL	A	1	6.642	-2.166	3.838	1.00	0.00	C
HETATM	119	H	MOL	A	1	6.256	-1.835	4.811	1.00	0.00	H
HETATM	120	C	MOL	A	1	6.787	-0.883	3.008	1.00	0.00	C
HETATM	121	H	MOL	A	1	7.529	-0.232	3.473	1.00	0.00	H
HETATM	122	H	MOL	A	1	7.137	-1.120	2.001	1.00	0.00	H
HETATM	123	C	MOL	A	1	5.468	-0.145	2.937	1.00	0.00	C
HETATM	124	C	MOL	A	1	5.074	0.675	4.012	1.00	0.00	C
HETATM	125	H	MOL	A	1	5.741	0.837	4.845	1.00	0.00	H
HETATM	126	C	MOL	A	1	3.797	1.261	4.016	1.00	0.00	C
HETATM	127	H	MOL	A	1	3.480	1.883	4.838	1.00	0.00	H
HETATM	128	C	MOL	A	1	2.923	1.036	2.941	1.00	0.00	C
HETATM	129	H	MOL	A	1	1.949	1.491	2.937	1.00	0.00	H
HETATM	130	C	MOL	A	1	3.316	0.229	1.863	1.00	0.00	C
HETATM	131	H	MOL	A	1	2.640	0.066	1.032	1.00	0.00	H
HETATM	132	C	MOL	A	1	4.585	-0.371	1.865	1.00	0.00	C
HETATM	133	H	MOL	A	1	4.870	-1.007	1.039	1.00	0.00	H
HETATM	134	C	MOL	A	1	5.829	-3.102	3.165	1.00	0.00	C
HETATM	135	O	MOL	A	1	4.893	-3.145	4.058	1.00	0.00	O
HETATM	136	N	MOL	A	1	5.892	-3.822	2.005	1.00	0.00	N
HETATM	137	H	MOL	A	1	6.783	-3.646	1.518	1.00	0.00	H
HETATM	138	C	MOL	A	1	5.554	-5.273	1.819	1.00	0.00	C
HETATM	139	H	MOL	A	1	4.347	-5.340	2.086	1.00	0.00	H
HETATM	140	C	MOL	A	1	5.905	-5.676	0.368	1.00	0.00	C
HETATM	141	H	MOL	A	1	6.958	-6.004	0.372	1.00	0.00	H
HETATM	142	H	MOL	A	1	5.288	-6.545	0.106	1.00	0.00	H
HETATM	143	C	MOL	A	1	5.694	-4.597	-0.690	1.00	0.00	C
HETATM	144	H	MOL	A	1	6.435	-3.783	-0.623	1.00	0.00	H

HETATM 145 H MOL A 1 5.854 -5.069 -1.677 1.00 0.00 H
HETATM 146 C MOL A 1 4.277 -4.024 -0.670 1.00 0.00 C
HETATM 147 O MOL A 1 3.318 -4.678 -0.186 1.00 0.00 O
HETATM 148 N MOL A 1 4.137 -2.811 -1.230 1.00 0.00 N
HETATM 149 H MOL A 1 3.174 -2.431 -1.434 1.00 0.00 H
HETATM 150 H MOL A 1 4.941 -2.336 -1.629 1.00 0.00 H
HETATM 151 C MOL A 1 5.296 -6.239 2.791 1.00 0.00 C
HETATM 152 O MOL A 1 4.948 -7.386 2.491 1.00 0.00 O
HETATM 153 N MOL A 1 2.387 -3.488 5.325 1.00 0.00 N
HETATM 154 H MOL A 1 3.294 -3.450 4.874 1.00 0.00 H
HETATM 155 C MOL A 1 1.244 -3.112 4.499 1.00 0.00 C
HETATM 156 H MOL A 1 0.872 -2.144 4.839 1.00 0.00 H
HETATM 157 C MOL A 1 1.747 -2.940 3.058 1.00 0.00 C
HETATM 158 H MOL A 1 2.248 -3.841 2.711 1.00 0.00 H
HETATM 159 H MOL A 1 0.918 -2.722 2.390 1.00 0.00 H
HETATM 160 H MOL A 1 2.453 -2.111 3.018 1.00 0.00 H
HETATM 161 C MOL A 1 0.077 -4.105 4.624 1.00 0.00 C
HETATM 162 O MOL A 1 -0.921 -3.798 5.273 1.00 0.00 O
HETATM 163 N MOL A 1 -3.704 -4.006 6.536 1.00 0.00 N
HETATM 164 H MOL A 1 -3.314 -4.931 6.668 1.00 0.00 H
HETATM 165 C MOL A 1 -4.655 -3.858 5.418 1.00 0.00 C
HETATM 166 H MOL A 1 -4.162 -3.281 4.639 1.00 0.00 H
HETATM 167 C MOL A 1 -4.905 -5.285 4.884 1.00 0.00 C
HETATM 168 H MOL A 1 -3.998 -5.604 4.372 1.00 0.00 H
HETATM 169 H MOL A 1 -5.043 -5.954 5.736 1.00 0.00 H
HETATM 170 C MOL A 1 -6.082 -5.549 3.934 1.00 0.00 C
HETATM 171 H MOL A 1 -5.953 -6.556 3.535 1.00 0.00 H
HETATM 172 H MOL A 1 -7.006 -5.548 4.511 1.00 0.00 H
HETATM 173 C MOL A 1 -6.261 -4.597 2.748 1.00 0.00 C
HETATM 174 H MOL A 1 -6.852 -5.116 1.994 1.00 0.00 H
HETATM 175 H MOL A 1 -6.848 -3.738 3.059 1.00 0.00 H
HETATM 176 N MOL A 1 -5.001 -4.161 2.130 1.00 0.00 N
HETATM 177 H MOL A 1 -4.424 -4.881 1.701 1.00 0.00 H
HETATM 178 C MOL A 1 -4.710 -2.924 1.793 1.00 0.00 C
HETATM 179 N MOL A 1 -5.236 -1.887 2.373 1.00 0.00 N
HETATM 180 H MOL A 1 -5.719 -2.031 3.262 1.00 0.00 H
HETATM 181 H MOL A 1 -4.815 -1.004 2.157 1.00 0.00 H
HETATM 182 N MOL A 1 -3.857 -2.689 0.855 1.00 0.00 N
HETATM 183 H MOL A 1 -3.287 -3.448 0.487 1.00 0.00 H
HETATM 184 H MOL A 1 -3.833 -1.779 0.391 1.00 0.00 H
HETATM 185 C MOL A 1 -5.932 -3.085 5.750 1.00 0.00 C
HETATM 186 O MOL A 1 -6.532 -3.286 6.828 1.00 0.00 O
HETATM 187 O MOL A 1 -6.379 -2.299 4.887 1.00 0.00 O
HETATM 188 C MOL A 1 -7.384 -4.383 -1.377 1.00 0.00 C
HETATM 189 O MOL A 1 -6.737 -3.928 -0.351 1.00 0.00 O
HETATM 190 N MOL A 1 -6.853 -5.001 -2.507 1.00 0.00 N
HETATM 191 H MOL A 1 -7.423 -5.819 -2.818 1.00 0.00 H
HETATM 192 C MOL A 1 -5.442 -5.480 -2.517 1.00 0.00 C
HETATM 193 H MOL A 1 -5.182 -5.741 -3.619 1.00 0.00 H
HETATM 194 C MOL A 1 -4.466 -4.367 -2.146 1.00 0.00 C
HETATM 195 H MOL A 1 -4.691 -4.115 -1.104 1.00 0.00 H
HETATM 196 H MOL A 1 -3.444 -4.761 -2.182 1.00 0.00 H
HETATM 197 C MOL A 1 -4.641 -3.148 -3.085 1.00 0.00 C

HETATM	198	H	MOL	A	1	-5.443	-3.335	-3.820	1.00	0.00	H
HETATM	199	H	MOL	A	1	-4.996	-2.282	-2.495	1.00	0.00	H
HETATM	200	C	MOL	A	1	-3.323	-2.867	-3.819	1.00	0.00	C
HETATM	201	H	MOL	A	1	-2.574	-2.415	-3.153	1.00	0.00	H
HETATM	202	H	MOL	A	1	-2.891	-3.822	-4.175	1.00	0.00	H
HETATM	203	N	MOL	A	1	-3.446	-2.012	-5.002	1.00	0.00	N
HETATM	204	H	MOL	A	1	-3.344	-2.449	-5.931	1.00	0.00	H
HETATM	205	C	MOL	A	1	-3.553	-0.685	-4.979	1.00	0.00	C
HETATM	206	N	MOL	A	1	-3.809	-0.048	-3.817	1.00	0.00	N
HETATM	207	H	MOL	A	1	-4.168	-0.523	-2.984	1.00	0.00	H
HETATM	208	H	MOL	A	1	-3.654	0.954	-3.675	1.00	0.00	H
HETATM	209	N	MOL	A	1	-3.380	0.066	-6.090	1.00	0.00	N
HETATM	210	H	MOL	A	1	-3.269	-0.519	-6.940	1.00	0.00	H
HETATM	211	H	MOL	A	1	-4.074	0.846	-6.168	1.00	0.00	H
HETATM	212	C	MOL	A	1	-5.482	-6.887	-2.394	1.00	0.00	C
HETATM	213	O	MOL	A	1	-5.821	-7.656	-3.301	1.00	0.00	O
HETATM	214	C	MOL	A	1	-2.759	-6.575	-4.137	1.00	0.00	C
HETATM	215	O	MOL	A	1	-2.137	-5.503	-4.557	1.00	0.00	O
HETATM	216	N	MOL	A	1	-2.656	-7.054	-2.830	1.00	0.00	N
HETATM	217	H	MOL	A	1	-3.165	-7.936	-2.676	1.00	0.00	H
HETATM	218	C	MOL	A	1	-1.435	-6.864	-1.971	1.00	0.00	C
HETATM	219	H	MOL	A	1	-1.055	-5.831	-2.124	1.00	0.00	H
HETATM	220	C	MOL	A	1	-1.809	-7.027	-0.446	1.00	0.00	C
HETATM	221	H	MOL	A	1	-2.400	-7.937	-0.283	1.00	0.00	H
HETATM	222	H	MOL	A	1	-0.881	-7.111	0.150	1.00	0.00	H
HETATM	223	C	MOL	A	1	-2.536	-5.799	0.226	1.00	0.00	C
HETATM	224	O	MOL	A	1	-2.083	-4.632	0.030	1.00	0.00	O
HETATM	225	O	MOL	A	1	-3.423	-6.156	1.039	1.00	0.00	O
HETATM	226	C	MOL	A	1	-0.549	-7.914	-2.424	1.00	0.00	C
HETATM	227	O	MOL	A	1	-0.156	-7.962	-3.594	1.00	0.00	O
HETATM	228	N	MOL	A	1	2.231	-7.512	-5.296	1.00	0.00	N
HETATM	229	H	MOL	A	1	1.287	-7.519	-4.921	1.00	0.00	H
HETATM	230	C	MOL	A	1	3.304	-7.016	-4.431	1.00	0.00	C
HETATM	231	H	MOL	A	1	4.045	-6.528	-5.064	1.00	0.00	H
HETATM	232	C	MOL	A	1	2.773	-5.925	-3.489	1.00	0.00	C
HETATM	233	H	MOL	A	1	1.995	-6.339	-2.849	1.00	0.00	H
HETATM	234	H	MOL	A	1	3.588	-5.601	-2.844	1.00	0.00	H
HETATM	235	C	MOL	A	1	2.234	-4.701	-4.212	1.00	0.00	C
HETATM	236	C	MOL	A	1	0.900	-4.670	-4.653	1.00	0.00	C
HETATM	237	H	MOL	A	1	0.262	-5.520	-4.479	1.00	0.00	H
HETATM	238	C	MOL	A	1	0.388	-3.538	-5.311	1.00	0.00	C
HETATM	239	H	MOL	A	1	-0.646	-3.520	-5.634	1.00	0.00	H
HETATM	240	C	MOL	A	1	1.212	-2.421	-5.524	1.00	0.00	C
HETATM	241	H	MOL	A	1	0.816	-1.533	-5.999	1.00	0.00	H
HETATM	242	C	MOL	A	1	2.554	-2.452	-5.107	1.00	0.00	C
HETATM	243	H	MOL	A	1	3.181	-1.581	-5.259	1.00	0.00	H
HETATM	244	C	MOL	A	1	3.061	-3.588	-4.450	1.00	0.00	C
HETATM	245	H	MOL	A	1	4.081	-3.587	-4.102	1.00	0.00	H
HETATM	246	C	MOL	A	1	4.074	-8.155	-3.731	1.00	0.00	C
HETATM	247	O	MOL	A	1	4.543	-7.990	-2.610	1.00	0.00	O
HETATM	248	N	MOL	A	1	-3.489	12.039	-2.647	1.00	0.00	N
HETATM	249	H	MOL	A	1	-3.689	12.392	-3.579	1.00	0.00	H
HETATM	250	C	MOL	A	1	-3.020	10.657	-2.528	1.00	0.00	C

```
HETATM 251 H MOL A 1 -2.451 10.572 -1.606 1.00 0.00 H
HETATM 252 C MOL A 1 -4.249 9.731 -2.410 1.00 0.00 C
HETATM 253 H MOL A 1 -4.753 9.962 -1.470 1.00 0.00 H
HETATM 254 H MOL A 1 -4.941 9.957 -3.219 1.00 0.00 H
HETATM 255 C MOL A 1 -3.930 8.226 -2.445 1.00 0.00 C
HETATM 256 H MOL A 1 -3.470 7.984 -3.397 1.00 0.00 H
HETATM 257 H MOL A 1 -3.226 7.974 -1.657 1.00 0.00 H
HETATM 258 C MOL A 1 -5.190 7.359 -2.296 1.00 0.00 C
HETATM 259 H MOL A 1 -5.935 7.689 -3.019 1.00 0.00 H
HETATM 260 H MOL A 1 -4.929 6.323 -2.513 1.00 0.00 H
HETATM 261 N MOL A 1 -5.747 7.432 -0.939 1.00 0.00 N
HETATM 262 H MOL A 1 -5.118 7.776 -0.211 1.00 0.00 H
HETATM 263 C MOL A 1 -6.966 7.104 -0.562 1.00 0.00 C
HETATM 264 N MOL A 1 -7.874 6.652 -1.377 1.00 0.00 N
HETATM 265 H MOL A 1 -7.736 6.654 -2.388 1.00 0.00 H
HETATM 266 H MOL A 1 -8.804 6.516 -1.014 1.00 0.00 H
HETATM 267 N MOL A 1 -7.336 7.220 0.676 1.00 0.00 N
HETATM 268 H MOL A 1 -6.697 7.585 1.380 1.00 0.00 H
HETATM 269 H MOL A 1 -8.300 7.003 0.906 1.00 0.00 H
HETATM 270 C MOL A 1 -2.056 10.294 -3.659 1.00 0.00 C
HETATM 271 O MOL A 1 -2.515 10.282 -4.824 1.00 0.00 O
HETATM 272 O MOL A 1 -0.865 10.064 -3.362 1.00 0.00 O
HETATM 273 O MOL A 1 2.486 -0.193 -2.852 1.00 0.00 O
HETATM 274 C MOL A 1 1.476 -0.763 -2.381 1.00 0.00 C
HETATM 275 N MOL A 1 1.542 -1.949 -1.724 1.00 0.00 N
HETATM 276 C MOL A 1 0.434 -2.592 -1.300 1.00 0.00 C
HETATM 277 N MOL A 1 0.560 -3.754 -0.646 1.00 0.00 N
HETATM 278 H MOL A 1 -0.306 -4.276 -0.425 1.00 0.00 H
HETATM 279 H MOL A 1 1.509 -4.158 -0.556 1.00 0.00 H
HETATM 280 C MOL A 1 -0.870 -2.000 -1.462 1.00 0.00 C
HETATM 281 H MOL A 1 -1.767 -2.516 -1.126 1.00 0.00 H
HETATM 282 C MOL A 1 -0.926 -0.756 -2.018 1.00 0.00 C
HETATM 283 H MOL A 1 -1.855 -0.201 -2.160 1.00 0.00 H
HETATM 284 N MOL A 1 0.206 -0.118 -2.459 1.00 0.00 N
HETATM 285 C MOL A 1 0.185 1.282 -2.952 1.00 0.00 C
HETATM 286 O MOL A 1 -1.170 1.739 -3.003 1.00 0.00 O
HETATM 287 H MOL A 1 0.623 1.315 -3.968 1.00 0.00 H
HETATM 288 C MOL A 1 0.962 2.196 -1.990 1.00 0.00 C
HETATM 289 H MOL A 1 1.864 1.705 -1.612 1.00 0.00 H
HETATM 290 H MOL A 1 1.238 3.140 -2.495 1.00 0.00 H
HETATM 291 S MOL A 1 -0.236 2.527 -0.652 1.00 0.00 S
HETATM 292 C MOL A 1 -1.415 2.771 -2.039 1.00 0.00 C
HETATM 293 H MOL A 1 -1.181 3.748 -2.501 1.00 0.00 H
HETATM 294 C MOL A 1 -2.850 2.748 -1.550 1.00 0.00 C
HETATM 295 H MOL A 1 -3.060 3.687 -1.015 1.00 0.00 H
HETATM 296 H MOL A 1 -3.011 1.898 -0.870 1.00 0.00 H
HETATM 297 O MOL A 1 -3.839 2.556 -2.584 1.00 0.00 O
HETATM 298 H MOL A 1 -3.717 3.156 -3.377 1.00 0.00 H
HETATM 299 O MOL A 1 2.399 1.285 -5.262 1.00 0.00 O
HETATM 300 H MOL A 1 2.535 2.263 -5.145 1.00 0.00 H
HETATM 301 H MOL A 1 2.596 0.918 -4.369 1.00 0.00 H
HETATM 302 O MOL A 1 -6.485 2.974 -1.912 1.00 0.00 O
HETATM 303 H MOL A 1 -5.531 3.164 -2.124 1.00 0.00 H
```

```
HETATM 304 H MOL A 1 -6.381 1.985 -2.095 1.00 0.00 H
HETATM 305 O MOL A 1 -6.722 4.077 -4.765 1.00 0.00 O
HETATM 306 H MOL A 1 -5.706 4.111 -4.797 1.00 0.00 H
HETATM 307 H MOL A 1 -6.792 3.758 -3.825 1.00 0.00 H
HETATM 308 O MOL A 1 2.461 -6.449 2.399 1.00 0.00 O
HETATM 309 H MOL A 1 2.662 -6.318 1.442 1.00 0.00 H
HETATM 310 H MOL A 1 3.365 -6.853 2.652 1.00 0.00 H
HETATM 311 O MOL A 1 -6.984 0.529 -4.226 1.00 0.00 O
HETATM 312 H MOL A 1 -7.606 1.225 -3.880 1.00 0.00 H
HETATM 313 H MOL A 1 -6.472 0.356 -3.356 1.00 0.00 H
HETATM 314 O MOL A 1 -5.400 1.918 -5.888 1.00 0.00 O
HETATM 315 H MOL A 1 -4.850 2.640 -5.438 1.00 0.00 H
HETATM 316 H MOL A 1 -5.767 1.445 -5.085 1.00 0.00 H
HETATM 317 H MOL A 1 0.342 5.675 5.988 1.00 0.00 H
HETATM 318 H MOL A 1 4.919 6.880 3.002 1.00 0.00 H
HETATM 319 H MOL A 1 -5.979 2.563 4.460 1.00 0.00 H
HETATM 320 H MOL A 1 -1.612 3.364 7.947 1.00 0.00 H
HETATM 321 H MOL A 1 -8.458 -4.269 -1.355 1.00 0.00 H
HETATM 322 H MOL A 1 -8.174 1.599 3.140 1.00 0.00 H
HETATM 323 H MOL A 1 -5.197 -7.318 -1.446 1.00 0.00 H
HETATM 324 H MOL A 1 -0.225 -8.670 -1.725 1.00 0.00 H
HETATM 325 H MOL A 1 2.421 -7.832 -6.224 1.00 0.00 H
HETATM 326 H MOL A 1 4.202 -9.103 -4.232 1.00 0.00 H
HETATM 327 H MOL A 1 5.404 -5.978 3.833 1.00 0.00 H
HETATM 328 H MOL A 1 -3.608 12.617 -1.840 1.00 0.00 H
HETATM 329 H MOL A 1 -4.622 5.294 4.796 1.00 0.00 H
HETATM 330 H MOL A 1 0.134 -5.071 4.144 1.00 0.00 H
HETATM 331 H MOL A 1 2.281 -3.767 6.279 1.00 0.00 H
HETATM 332 H MOL A 1 -3.459 -3.238 7.128 1.00 0.00 H
HETATM 333 H MOL A 1 5.272 4.794 5.288 1.00 0.00 H
HETATM 334 H MOL A 1 8.893 4.592 2.116 1.00 0.00 H
HETATM 335 H MOL A 1 -3.382 -7.115 -4.834 1.00 0.00 H
HETATM 336 H MOL A 1 8.139 -3.291 4.908 1.00 0.00 H
END
```

Table S1. Descriptors of intra- and intermolecular interactions in polymorph II of *Lamivudine*.

	rho	d2rho	g	v	h	E
3 (3,-1) S(1) 1 - S(1) 28	0,015633	0,027051	0,007315	-0,007867	-0,00055	-10,3422131
6 (3,-1) H(8B) 26 - H(1) 51	0,004832	0,021991	0,004062	-0,002626	0,001436	-3,45222469
15 (3,-1) O(1) 2 - H(3A)	0,008381	0,041513	0,007912	-0,005445	0,002466	-7,15817343
16 (3,-1) O(1) 2 - O(3)	0,003013	0,010444	0,001921	-0,001231	0,00069	-1,61831249
22 (3,-1) O(2) 3 - H(7)	0,013837	0,07623	0,014995	-0,010932	0,004063	-14,3715614
25 (3,-1) O(2) 3 - H(3A)	0,013278	0,080081	0,015485	-0,010949	0,004536	-14,3939102
27 (3,-1) O(2) 3 - O(2)	0,005062	0,02368	0,004375	-0,00283	0,001545	-3,7204097
26 (3,-1) H(2) 17 - N(2)	0,03946	0,151543	0,038389	-0,038893	-0,0005	-51,129998
39 (3,-1) O(3) 4 - H(3)	0,016549	0,084023	0,017089	-0,013173	0,003916	-17,3176526
40 (3,-1) O(3) 4 - H(3B)	0,016507	0,090021	0,018076	-0,013647	0,004429	-17,9407884
41 (3,-1) O(3) 4 - H(6)	0,006812	0,030698	0,005819	-0,003964	0,001855	-5,21120284
42 (3,-1) O(3) 4 - H(2B)	0,005534	0,022637	0,00427	-0,002881	0,001389	-3,78745595
43 (3,-1) O(3) 4 - H(8A)	0,004232	0,020154	0,003677	-0,002315	0,001362	-3,04337401
44 (3,-1) O(3) 4 - H(8B)	0,009332	0,039552	0,00778	-0,005671	0,002108	-7,45528035
49 (3,-1) O(3) 4 - O(1)	0,003013	0,010444	0,001921	-0,001231	0,00069	-1,61831249
61 (3,-1) C(7) 14 - H(3)	0,002787	0,010672	0,001937	-0,001206	0,000731	-1,58544668
65 (3,-1) N(2) 6 - H(2)	0,03946	0,151543	0,038389	-0,038893	-0,0005	-51,129998
73 (3,-1) H(3B) 22 - O(3)	0,016507	0,090022	0,018076	-0,013647	0,004429	-17,9407884
74 (3,-1) H(3A) 21 - O(2)	0,013278	0,080082	0,015485	-0,010949	0,004536	-14,3939102
75 (3,-1) H(3A) 21 - H(7)	0,004002	0,018406	0,003357	-0,002113	0,001244	-2,77781827
94 (3,-1) H(2B) 19 - H(3)	0,00549	0,026089	0,004839	-0,003155	0,001683	-4,14766523
95 (3,-1) H(2A) 18 - H(2A)	0,004251	0,014868	0,002798	-0,001879	0,000919	-2,47019428
102 (3,-1) H(3) 20 - H(2B)	0,00549	0,026089	0,004839	-0,003155	0,001683	-4,14766523
105 (3,-1) C(4) 11 - H(2B)	0,00551	0,019069	0,003672	-0,002576	0,001095	-3,38649307
112 (3,-1) H(6) 23 - O(3)	0,006812	0,030698	0,005819	-0,003964	0,001855	-5,21120284
119 (3,-1) H(7) 24 - H(3A)	0,004002	0,018407	0,003358	-0,002113	0,001244	-2,77781827
126 (3,-1) H(8B) 26 - O(3)	0,009332	0,039552	0,00778	-0,005671	0,002108	-7,45528035
133 (3,-1) H(2B) 19 - O(3)	0,005534	0,022637	0,00427	-0,002881	0,001389	-3,78745595
135 (3,-1) H(2B) 19 - C(4)	0,00551	0,019069	0,003672	-0,002576	0,001095	-3,38649307
137 (3,-1) H(3) 20 - C(7)	0,002787	0,010672	0,001937	-0,001206	0,000731	-1,58544668
147 (3,-1) H(6) 23 - H(8A)	0,000993	0,005471	0,00094	-0,000513	0,000428	-0,67440642
150 (3,-1) H(8A) 25 - H(6)	0,000993	0,005471	0,00094	-0,000513	0,000428	-0,67440642
153 (3,-1) H(2B) 26 - N(3)	0,003628	0,019563	0,003506	-0,002122	0,001384	-2,78964996
(3,-1) N(3)-H(2B)	0,003628	0,019563	0,003506	-0,002122	0,001384	-2,78964996
(3,-1) S(1) 1 - C(5)	0,005927	0,014773	0,003019	-0,002346	0,000674	-3,08412762
(3,-1) C(5) - S(1)	0,005927	0,014773	0,003019	-0,002346	0,000674	-3,08412762

Table S2. Descriptors of intra- and intermolecular interactions in complexes A and B.

Complex A

Connected atoms	Residues	Density of all electrons	Hamiltonian kinetic energy K(r)	Potential energy density	Energy density E(r) or H(r)	Laplacian of electron density	Ellipticity of electron density
304O--247H	ASP100--3TC92	0,046	0,002	-0,034	-0,002	0,123	0,055
199H--250H	ARG54--3TC92	0,003	-0,001	-0,001	0,001	0,014	1,533
247H--246N	3TC92--3TC92	0,305	0,445	-0,494	-0,445	-1,582	0,035
67H--249C	TRP25--3TC92	0,006	-0,001	-0,003	0,001	0,020	0,776
281H--250H	ARG95--3TC92	0,001	0,000	0,000	0,000	0,003	0,218
246N--248H	3TC92--3TC92	0,321	0,471	-0,520	-0,471	-1,691	0,035
248H--163O	3TC92--GLN47	0,027	0,001	-0,020	-0,001	0,068	0,030
250H--249C	3TC92--3TC92	0,276	0,281	-0,325	-0,281	-0,943	0,037
246N--313H	3TC92--PHE104	0,006	-0,001	-0,003	0,001	0,018	0,162
246N--245C	3TC92--3TC92	0,342	0,553	-0,848	-0,553	-1,031	0,171
249C--245C	3TC92--3TC92	0,284	0,259	-0,342	-0,259	-0,708	0,169
69H--251C	TRP25--3TC92	0,008	-0,001	-0,004	0,001	0,026	1,265
31O--265H	GLU20--3TC92	0,007	-0,001	-0,004	0,001	0,024	0,116
249C--251C	3TC92--3TC92	0,323	0,342	-0,464	-0,342	-0,877	0,322
245C--244N	3TC92--3TC92	0,332	0,481	-0,685	-0,481	-1,108	0,116
251C--252H	3TC92--3TC92	0,283	0,294	-0,329	-0,294	-1,034	0,023
69H--260S	TRP25--3TC92	0,006	-0,001	-0,002	0,001	0,016	0,139
251C--318C	3TC92--PHE104	0,008	-0,001	-0,004	0,001	0,025	1,617
244N--165H	3TC92--GLN47	0,047	0,004	-0,032	-0,004	0,097	0,049
252H--265H	3TC92--3TC92	0,007	-0,001	-0,004	0,001	0,025	0,363
251C--253N	3TC92--3TC92	0,308	0,477	-0,750	-0,477	-0,813	0,073
244N--315C	3TC92--PHE104	0,005	-0,001	-0,003	0,001	0,016	1,040
265H--263C	3TC92--3TC92	0,274	0,273	-0,313	-0,273	-0,930	0,042
334O--264H	WAT158--3TC92	0,020	0,001	-0,014	-0,001	0,050	0,157

244N--243C	3TC92--3TC92	0,335	0,478	-0,675	-0,478	-1,120	0,177
40H--260S	VAL22--3TC92	0,006	-0,001	-0,003	0,001	0,022	0,345
252H--255O	3TC92--3TC92	0,017	-0,002	-0,013	0,002	0,069	1,559
288H--266O	ARG95--3TC92	0,046	0,003	-0,033	-0,003	0,114	0,030
253N--243C	3TC92--3TC92	0,282	0,356	-0,508	-0,356	-0,810	0,145
264H--263C	3TC92--3TC92	0,280	0,287	-0,324	-0,287	-1,001	0,030
263C--266O	3TC92--3TC92	0,239	0,290	-0,452	-0,290	-0,512	0,013
253N--320C	3TC92--PHE104	0,007	-0,001	-0,004	0,001	0,021	1,801
243C--324C	3TC92--PHE104	0,005	-0,001	-0,002	0,001	0,018	1,444
243C--242O	3TC92--3TC92	0,379	0,644	-1,227	-0,644	-0,246	0,132
145H--242O	PHE46--3TC92	0,005	-0,001	-0,003	0,001	0,020	0,778
263C--261C	3TC92--3TC92	0,258	0,213	-0,273	-0,213	-0,614	0,029
267H--266O	3TC92--3TC92	0,304	0,464	-0,537	-0,464	-1,564	0,024
253N--254C	3TC92--3TC92	0,253	0,271	-0,379	-0,271	-0,652	0,144
267H--239O	3TC92--GLU73	0,055	0,004	-0,044	-0,004	0,146	0,012
260S--261C	3TC92--3TC92	0,169	0,107	-0,156	-0,107	-0,232	0,097
260S--47H	3TC92--VAL22	0,007	-0,001	-0,003	0,001	0,024	1,186
255O--321H	3TC92--PHE104	0,008	-0,001	-0,005	0,001	0,029	0,837
261C--255O	3TC92--3TC92	0,252	0,332	-0,529	-0,332	-0,542	0,070
260S--257C	3TC92--3TC92	0,172	0,112	-0,163	-0,112	-0,247	0,070
255O--254C	3TC92--3TC92	0,260	0,354	-0,562	-0,354	-0,581	0,076
242O--147H	3TC92--PHE46	0,008	-0,001	-0,005	0,001	0,030	0,125
258H--257C	3TC92--3TC92	0,278	0,283	-0,323	-0,283	-0,972	0,010
254C--257C	3TC92--3TC92	0,247	0,197	-0,256	-0,197	-0,550	0,017
266O--12H	3TC92--ILE6	0,005	-0,001	-0,003	0,001	0,019	0,295
322C--256H	PHE104--3TC92	0,007	-0,001	-0,003	0,001	0,023	1,304
261C--262H	3TC92--3TC92	0,279	0,283	-0,318	-0,283	-0,987	0,034
254C--256H	3TC92--3TC92	0,286	0,299	-0,331	-0,299	-1,067	0,024
242O--85H	3TC92--LEU32	0,003	-0,001	-0,002	0,001	0,014	0,572
266O--8H	3TC92--ILE6	0,007	-0,001	-0,005	0,001	0,026	0,270

258H--85H	3TC92--LEU32	0,007	-0,001	-0,003	0,001	0,023	0,140
257C--91H	3TC92--LEU32	0,005	-0,001	-0,002	0,001	0,018	0,563
260S--333H	3TC92--WAT143	0,019	0,000	-0,012	0,000	0,047	0,047
266O--15H	3TC92--ILE6	0,003	-0,001	-0,001	0,001	0,012	2,962
257C--259H	3TC92--3TC92	0,276	0,280	-0,318	-0,280	-0,968	0,014
242O--330H	3TC92--WAT137	0,027	0,000	-0,020	0,000	0,077	0,038
255O--14C	3TC92--ILE6	0,002	-0,001	-0,001	0,001	0,010	6,311
262H--235H	3TC92--GLU73	0,002	0,000	-0,001	0,000	0,005	0,247
262H--331O	3TC92--WAT143	0,009	-0,001	-0,006	0,001	0,032	0,430
256H--328O	3TC92--WAT137	0,009	-0,001	-0,006	0,001	0,029	0,355
262H--15H	3TC92--ILE6	0,003	-0,001	-0,001	0,001	0,008	0,196
259H--331O	3TC92--WAT143	0,010	-0,001	-0,007	0,001	0,038	0,115

Complex B

Connected atoms	Residues	Density of all electrons	Hamiltonian kinetic energy K(r)	Potential energy density	Energy density E(r) or H(r)	Laplacian of electron density	Ellipticity of electron density
146O--293H	GLN28--3TC60	0,031	0,001	-0,022	-0,001	0,080	0,028
293H--291N	3TC60--3TC60	0,320	0,471	-0,520	-0,471	-1,690	0,035
223O--292H	ASP42--3TC60	0,047	0,001	-0,035	-0,001	0,129	0,041
292H--291N	3TC60--3TC60	0,301	0,437	-0,486	-0,437	-1,554	0,036
158H--291N	ALA31--3TC60	0,002	0,000	-0,001	0,000	0,008	0,130
148H--289N	GLN28--3TC60	0,047	0,004	-0,031	-0,004	0,096	0,055
232H--289N	PHE46--3TC60	0,008	-0,001	-0,004	0,001	0,026	0,233
291N--290C	3TC60--3TC60	0,341	0,546	-0,828	-0,546	-1,055	0,171
290C--289N	3TC60--3TC60	0,334	0,488	-0,697	-0,488	-1,114	0,120
233H--287O	PHE46--3TC60	0,005	-0,001	-0,003	0,001	0,018	0,516
290C--294C	3TC60--3TC60	0,287	0,264	-0,347	-0,264	-0,722	0,162
234C--288C	PHE46--3TC60	0,006	-0,001	-0,003	0,001	0,021	469,764
289N--288C	3TC60--3TC60	0,328	0,448	-0,629	-0,448	-1,069	0,167
50H--294C	TRP15--3TC60	0,003	-0,001	-0,001	0,001	0,009	0,133
295H--294C	3TC60--3TC60	0,278	0,284	-0,327	-0,284	-0,962	0,035

130H--287O	PHE27--3TC60	0,012	-0,001	-0,008	0,001	0,038	0,041
294C--237C	3TC60--PHE46	0,006	-0,001	-0,003	0,001	0,016	3,251
288C--287O	3TC60--3TC60	0,387	0,661	-1,278	-0,661	-0,174	0,148
288C--128H	3TC60--PHE27	0,005	-0,001	-0,002	0,001	0,020	0,893
294C--296C	3TC60--3TC60	0,324	0,344	-0,467	-0,344	-0,881	0,320
288C--298N	3TC60--3TC60	0,279	0,349	-0,502	-0,349	-0,787	0,135
295H--205N	3TC60--ARG37	0,007	-0,001	-0,004	0,001	0,023	0,563
287O--315H	3TC60--WAT61	0,013	-0,001	-0,010	0,001	0,045	0,097
296C--298N	3TC60--3TC60	0,311	0,485	-0,769	-0,485	-0,801	0,078
128H--303H	PHE27--3TC60	0,008	-0,002	-0,004	0,002	0,031	0,367
296C--52H	3TC60--TRP15	0,003	-0,001	-0,001	0,001	0,008	1,171
296C--297H	3TC60--3TC60	0,286	0,301	-0,339	-0,301	-1,048	0,026
298N--299C	3TC60--3TC60	0,253	0,265	-0,369	-0,265	-0,646	0,155
297H--205N	3TC60--ARG37	0,012	-0,001	-0,007	0,001	0,037	0,223
241C--301H	PHE46--3TC60	0,004	-0,001	-0,002	0,001	0,012	0,104
303H--302C	3TC60--3TC60	0,279	0,287	-0,324	-0,287	-1,002	0,012
52H--305S	TRP15--3TC60	0,009	-0,001	-0,004	0,001	0,026	0,017
313O--301H	WAT61--3TC60	0,017	0,000	-0,012	0,000	0,046	0,053
301H--299C	3TC60--3TC60	0,286	0,304	-0,334	-0,304	-1,094	0,026
297H--300O	3TC60--3TC60	0,017	-0,002	-0,013	0,002	0,067	1,367
299C--302C	3TC60--3TC60	0,249	0,199	-0,260	-0,199	-0,557	0,017
69H--305S	LEU18--3TC60	0,004	-0,001	-0,002	0,001	0,014	0,241
297H--310H	3TC60--3TC60	0,011	-0,002	-0,007	0,002	0,045	0,728
299C--300O	3TC60--3TC60	0,249	0,337	-0,553	-0,337	-0,487	0,101
273O--304H	GLU55--3TC60	0,007	-0,001	-0,004	0,001	0,027	1,768
302C--305S	3TC60--3TC60	0,172	0,111	-0,161	-0,111	-0,245	0,079
302C--304H	3TC60--3TC60	0,279	0,285	-0,325	-0,285	-0,979	0,019
207H--311O	ARG37--3TC60	0,037	0,002	-0,028	-0,002	0,098	0,066
310H--308C	3TC60--3TC60	0,278	0,280	-0,317	-0,280	-0,972	0,038
304H--277H	3TC60--GLU56	0,009	-0,001	-0,005	0,001	0,032	0,093

300O--312H	3TC60--3TC60	0,031	0,001	-0,027	-0,001	0,102	0,106
15O--308C	GLU10--3TC60	0,003	-0,001	-0,002	0,001	0,014	1,181
305S--306C	3TC60--3TC60	0,174	0,116	-0,167	-0,116	-0,260	0,118
300O--306C	3TC60--3TC60	0,237	0,282	-0,444	-0,282	-0,485	0,056
312H--311O	3TC60--3TC60	0,339	0,544	-0,617	-0,544	-1,889	0,024
305S--73H	3TC60--LEU18	0,007	-0,001	-0,003	0,001	0,021	0,090
308C--306C	3TC60--3TC60	0,249	0,197	-0,254	-0,197	-0,565	0,050
308C--311O	3TC60--3TC60	0,236	0,303	-0,494	-0,303	-0,447	0,029
304H--282H	3TC60--GLU56	0,009	-0,002	-0,005	0,002	0,033	0,539
300O--282H	3TC60--GLU56	0,005	-0,001	-0,003	0,001	0,020	0,345
308C--309H	3TC60--3TC60	0,279	0,283	-0,321	-0,283	-0,983	0,037
306C--307H	3TC60--3TC60	0,279	0,285	-0,319	-0,285	-1,008	0,031
307H--282H	3TC60--GLU56	0,006	-0,001	-0,003	0,001	0,024	1,063
311O--324H	3TC60--WAT71	0,035	0,001	-0,027	-0,001	0,095	0,034
307H--286O	3TC60--GLU56	0,013	0,000	-0,008	0,000	0,032	0,018
309H--23H	3TC60--VAL12	0,000	0,000	0,000	0,000	0,000	1,145

Table S3. Descriptors of molecular VP in crystals and complexes.

Refcode	Molecule	Vmol	Smol	$S_{S/H}$	$S_{S/O}$	$S_{S/C}$	$S_{O/H}$	$S_{C/H}$	$S_{H/H}$	$S_{H/N}$	$S_{C/O}$	$S_{C/C}$	$S_{C/N}$	$S_{N/O}$	$S_{N/N}$	$S_{O/O}$	$S_{S/S}$	$S_{S/N}$	$S_{H/Cl}$	$S_{Cl/Y}$
COWSUD	3TC	235.12	260.14	21.34	7.88	0.90	95.45	13.90	80.61	10.50	3.66	6.90	6.19	5.54	0.80	6.42	0.00	0.00		
	H3TC(+)	254.55	272.25	26.94	5.26		127.19	18.66	58.49	12.56	6.21	6.60	2.68	5.23		2.43	0.00	0.00		
COWTAK	3TC	246.50	268.60	28.47	0.87	0.00	70.05	23.57	106.50	26.80	4.96	1.23	3.63	0.80	1.11		0.61	0.00		
IGAVEU	H3TC(+)	245.82	262.61	22.02			111.36	11.67	91.12	11.58	2.20	5.53	1.45	2.19	0.02	3.03		0.43		
	H3TC(+)	245.89	264.60	21.64	2.34		109.05	12.17	93.70	12.93	2.47	5.53	1.45	2.22	0.02	0.64		0.43		
LASZAI	H3TC(+)	248.39	265.17	19.00	2.46	2.24	51.36	20.49	103.52	8.77	7.38			4.69		0.17		5.39	35.19	4.5
LASZEM01	H3TC(+)	250.87	265.80	29.04	0.18	1.85	60.14	21.66	103.04	11.45	6.43			5.23		0.20		0.57	19.64	6.38
LICVEB	3TC	260.23	273.61	17.59		0.05	76.75	12.84	90.42	13.09	1.16	7.97	6.61	2.12	2.99	3.97	0.63		29.35	8.06
	3TC	270.63	284.52	20.85		0.44	71.62	14.57	94.32	14.67	1.99	7.28	7.25	2.51	2.58	3.75	1.03		33.52	8.13
	H3TC(+)	252.47	275.86	22.78	3.10	0.96	82.15	11.21	78.00	13.31	2.06	7.28	7.25	2.53	2.58	1.67	1.03		33.65	6.3
	H3TC(+)	254.62	274.98	24.59	0.79		79.23	10.25	85.50	13.90	1.61	7.97	6.61	2.13	2.99	3.39	0.63		32.20	3.17
LIGWUV	3TC	249.03	270.06	31.78	1.19		68.78	14.28	98.98	12.08	2.34	6.87	5.56	2.15	5.57	1.27			18.61	0.59
	3TC	249.95	275.67	18.44	2.96		74.01	13.61	117.95	12.37	2.40	6.88	5.56	1.27	5.57	2.89			11.14	0.62
	H3TC(+)	265.40	278.64	41.24	4.50	0.20	51.41	22.21	108.10	16.63	2.40	0.85	3.19	1.12	1.40	2.88	0.44	0.63	20.59	0.83
	H3TC(+)	252.23	273.39	29.96	0.26		73.89	22.01	105.89	17.14	2.49	0.85	3.19	2.01	1.40	1.13	0.44		11.07	1.67
NUWKEX	3TC	242.44	265.52	15.68		0.17	56.11	62.37	100.61	22.53	2.54	1.86	2.50	1.14		0.01				
QUWXOW	3TC	230.48	262.84	31.35	3.26	1.43	58.41	25.77	113.63	25.35	0.41					0.13		3.11		
	3TC	233.34	259.91	21.23	2.02	8.87	66.93	24.19	94.47	31.94	1.77	0.55	3.88	1.03		0.14		2.87		
	3TC	237.64	265.83	18.59	0.34	6.45	65.40	32.12	103.25	31.38	1.77	0.55	3.88	1.03				1.05		
	3TC	239.85	260.70	34.94	3.02	4.10	62.80	25.41	101.02	25.49	0.98			0.93				2.01		
RIBFIT	H3TC(+)	255.03	269.56	22.93		0.85	86.92	30.22	95.70	9.65	13.40	0.49	1.74	5.31	0.35	1.99				
	H3TC(+)	253.52	271.24	21.64		1.16	87.95	31.39	96.07	9.67	14.19	0.11	1.38	5.14	0.44	2.09				
	H3TC(+)	253.42	276.90	20.67	1.65	1.51	87.06	29.90	101.20	9.38	12.91	0.93	1.44	6.33	0.07	3.85				
	H3TC(+)	259.89	277.05	20.49	2.03	1.34	91.10	30.16	98.67	9.88	10.63	0.97	0.30	7.27		4.22				
RIBFOZ	H3TC(+)	261.55	270.48	14.44	3.41		102.50	19.39	63.26	3.35	0.26	9.60	6.49	0.02	1.63	3.90	0.18	34.10	7.95	
	H3TC(+)	264.50	268.76	14.41	9.80		97.39	17.92	59.24	3.41	1.87	10.20	7.07	0.02	1.63	1.07	0.18	35.59	8.98	
RUKHAG	3TC	239.01	268.61	17.62		12.08	61.43	24.01	104.16	33.99	2.54			1.76		1.52	3.48	6.01		
RUKHAG01	3TC	237.05	257.23	21.92	0.23	1.07	63.38	22.10	103.86	34.78	2.11	3.62	0.89	1.62		0.53	1.14			

	3TC	242.99	259.14	28.29	5.13	7.75	53.78	20.18	98.61	30.73	1.71	3.62	0.95	3.97		0.28	1.14	3.00		
UYEYEE	H3TC(+)	243.4	262.42	18.61	3.40	0.40	100.15	23.40	90.56	8.76	8.45	0.45	0.88	6.80		0.55				
VAWPIT	H3TC(+)	239.67	263.31	28.89	1.66		87.70	22.29	81.60	16.40	9.82	1.22	4.21	6.42	0.48	1.69	0.90			
VISVOK	H3TC(+)	266.84	273.39	23.63	4.68	3.73	110.15	23.12	70.65	12.42	7.91	3.12	3.72	1.10	0.06	2.76	4.24	2.10		
	H3TC(+)	261.03	279.72	20.67	0.80	2.31	103.97	20.86	85.27	12.60	11.94	2.42	3.25	1.94	0.34	11.27		2.10		
VISVUQ	H3TC(+)	255.75	264.04	25.21	1.08	3.35	87.07	21.76	86.80	13.22	10.36	3.35	0.41	6.61		2.82	2.02			
	H3TC(+)	244.33	265.78	25.04	2.84	2.86	95.46	23.82	89.65	12.76	5.50			3.48	0.06	2.02	2.29			
VISWAX	3TC	232.91	266.03	24.41	1.91		74.77	11.58	111.48	18.59	1.53	8.30	7.20	0.01	2.07	4.18				
	3TC	233.44	255.56	16.69	3.50	1.02	77.90	12.06	102.05	19.30	1.54	8.30	7.20	0.01	2.07	3.92				
WOMHEM	3TC	236.98	266.75	29.12	0.45		72.75	11.75	105.27	30.65	0.05		8.27	1.86	4.25	0.04		2.29		
modelA	3TC	248.73	281.83	16.83			84.81	18.12	133.94	17.07	0.24	7.63	3.08			0.1				
modelB	3TC	299.88	322.75	19.24	3.65		63.41	19.48	182.42	21.88	1.29	5.3	3.99	1.49		0.61				
4QWD	3TC-3TPP	526.84	500.19	12.75	3.13		204.12	24.34	163.68	28.08	4.38			4.86		31.94				
5TB8	3TC-3TPP	488.6	483.32	13.47	1.01	1.76	213.71	29.93	129.15	26.57	2.49			2.94		43.62				
5TBC	3TC-3TPP	547.05	522.49	12.79	2.9		210.22	25.2	148.65	15.9	6.61	1.62	1.38	16.62	1.09	63.95				
	3TC-1TPP	399.2	398.21	22.53			131.6	22.84	132.88	11.82	9.12	1.59	5.18	6.35	8.44	38.93				
